# Advances in the polymeric delivery of nucleic acid vaccines

**DOI:** 10.7150/thno.70853

**Published:** 2022-05-13

**Authors:** Gang Chen, Bowen Zhao, Elena F. Ruiz, Fuwu Zhang

**Affiliations:** 1 School of Rehabilitation Science and Engineering, University of Health and Rehabilitation Sciences, Qingdao 266024, PR China; 2 Department of Chemistry, University of Miami, Coral Gables, FL 33146, United States; 3 The Dr. John T. Macdonald Foundation Biomedical Nanotechnology Institute, University of Miami, Miami, FL 33136, United States

**Keywords:** nucleic acid vaccine, polymer, gene delivery, humoral immunity, cellular immunity

## Abstract

Nucleic acid vaccines, especially messenger RNA (mRNA) vaccines, display unique benefits in the current COVID-19 pandemic. The application of polymeric materials as delivery carriers has greatly promoted nucleic acid vaccine as a promising prophylactic and therapeutic strategy. The inherent properties of polymeric materials render nucleic acid vaccines with excellent in vivo stability, enhanced biosafety, specific cellular uptake, endolysosomal escape, and promoted antigen expression. Although polymeric delivery of nucleic acid vaccines has progressed significantly in the past decades, clinical translation of polymer-gene vaccine systems still faces insurmountable challenges. This review summarizes the diverse polymers and their characterizations and representative formulations for nucleic acid vaccine delivery. We also discussed existing problems, coping strategies, and prospect relevant to applications of nucleic acid vaccines and polymeric carriers. This review highlights the rational design and development of polymeric vaccine delivery systems towards meeting the goals of defending serious or emerging diseases.

## Introduction

Infectious diseases, for instance, coronavirus disease 2019 (COVID-19), tumors, and immune diseases are serious threats to public safety and health. The recurrence, spread, transmission, and long course of diseases bring great obstacles to the treatment process 1. Effective vaccines against such severe diseases are urgently needed to control the significant mortality and morbidity 2. Single-dose immunization is the center of vaccine design due to convenience and cost-effectiveness, yet drawbacks such as low immune efficiency and residual toxicity in traditional vaccines including live-attenuated, inactivated, or subunit vaccines, put obstacles in the path of vaccine design 3. With the innovation of gene editing technology, nucleic acid vaccines including DNA and RNA vaccines occupy the dominant position in the field of vaccines with their advantageous safety, easy construction, and rapid scalable production. Recently, a variety of messenger RNA (mRNA) vaccines have been developed against COVID-19, representing a milestone in nucleic acid vaccine development 4.

Nucleic acid vaccines induce strong T helper cell (Th)1 and cluster of differentiation (CD)8^+^ immune responses by introducing exogenous genes and expressing antigens in the host cells, which is suitable for the treatment of metastatic, long-term, and recurring diseases 5. Meanwhile, nucleic acid vaccines are subject to many limitations in the application process, such as the degradation by nucleases, rapid clearance by the reticular endothelial system, and poor uptake and translation efficiencies, which often leads to failures in inducing the expected immune responses 6. Although researchers have devoted their efforts to improve the immunogenicity of naked nucleic acid vaccines, such as through the use of more potent promoter/enhancer systems, optimization of antigen-coding sequences, and amelioration of immunization routes, naked DNA or RNA vaccines still exhibit limited elevation in triggering the host immune responses 7. Therefore, it is necessary to develop suitable delivery carriers for nucleic acid vaccines toward improved immune potency.

Initially, lentiviruses and adenoviruses were used as biological vectors in nucleic acid vaccines, improving the transfection efficiency to a certain extent. However, such vectors still have defects: (1) they cannot be expressed continuously, (2) they are not easily modified, and (3) they carry potential risks associated with transfection 8. Recently, lipid nanoparticle (LNP)-mRNA vaccines have been approved for emergency use to prevent COVID-19. Two mRNA-LNP vaccines, mRNA-1273 and BNT162b2, have been developed and approved by Food and Drug Administration (FDA), and following several others are in the clinical evaluation stage. LNP-based delivery vehicles have shown remarkable prospects in the development of COVID-19 vaccines and other nanomedicines 9, 10. Despite having less clinical advances than LNPs, polymeric carriers offer similar advantages and have been developed for the delivery of nucleic acid vaccines with the innovation of technology. Polymeric carriers have a good nucleic acid loading efficiency, improving the stability of nucleic acids and avoiding their degradation by overcoming the obstacles of cellular internalization, and improving the efficiency of disease prevention and treatment 11. The diversity and customizability of polymer carriers further improve their therapeutic efficiencies by regulating the release of nucleic acids, long cycle properties, tissue or cell targeting, and stimulus responsiveness. These advantages of polymer carriers make it an attractive choice for nucleic acid vaccine delivery. Nucleic acid vaccines and delivery systems are the most significant factors in regulating transfection efficiency and antigen expression levels, which fundamentally determines the design idea of vaccines and their fate in vivo.

**Figure 1:**
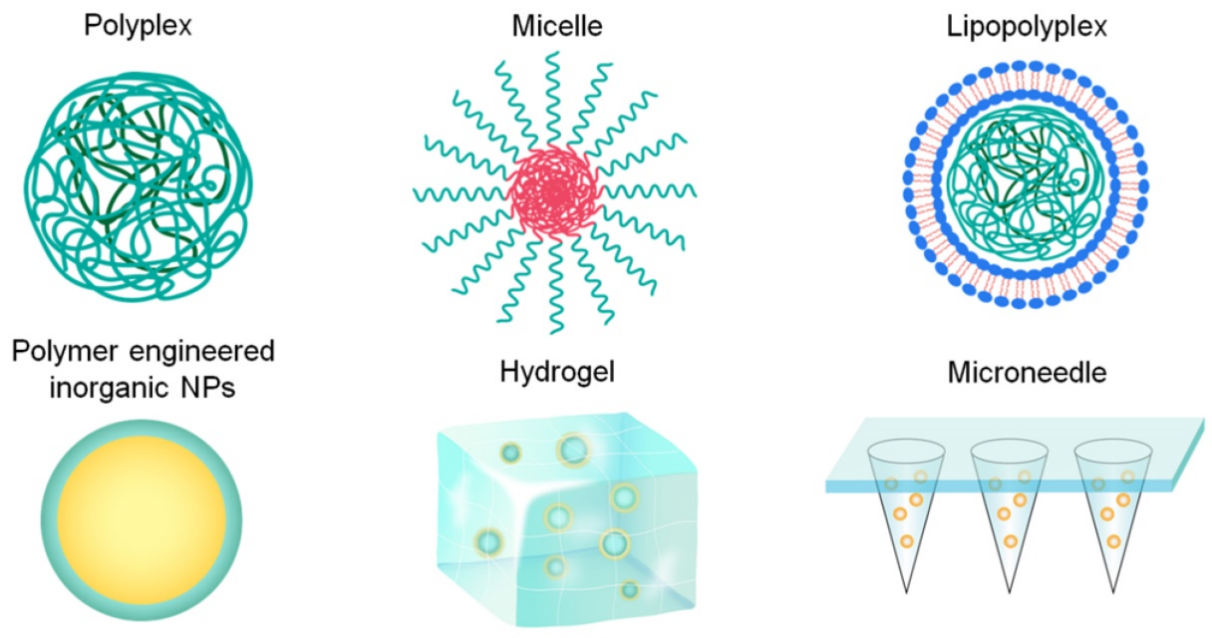
Representative polymeric formulations used for vaccine delivery including polyplex, micelle, lipopolyplex, polymer engineered inorganic nanoparticles (NPs), hydrogel, and microneedle.

In this review, we summarize the types and characteristics of polymer carriers and formulations for nucleic acid vaccine delivery in detail (Figure 1). Furthermore, we discuss the challenges relevant to nucleic acid vaccines and their delivery using polymer-based systems. Finally, we summarize the strategies for enhancing the efficacy of nucleic acid vaccines in the development of polymeric delivery systems.

## 2. Types of polymer carriers for nucleic acid vaccine delivery

An appropriate vaccine delivery system to guide the in vivo process is indispensable for efficient vaccine potency due to the hydrophilicity, limited biological stability, easy degradation by nucleases or lysosomes, and poor pharmacokinetics of naked nucleic acid vaccines. The presence of vaccine carriers reduces the degradation of nucleic acids in the body and offers a variety of attractive characteristics that can potentially improve vaccine potency.

Lipid- and polymer-based delivery systems are the most widely developed for vaccine delivery. Lipid-based delivery systems have good biocompatibility and can protect nucleic acids from degradation by nuclease and achieve endocytosis of nucleic acids to improve the transfection efficiency. Lipid-related surface modifications also confer characteristics, such as long cycling and antigen-presenting cell (APC) targeting 12. On the other hand, polymer-based carrier delivery systems, which may be precisely customized by introducing beneficial end groups, are increasingly promising 13. Initially, cationic polymers consisting of a few repeating units, such as polylysine and polyornithine, were used as the carriers, where positive charges from the polymer bind to the negatively charged nucleic acids, enhancing cell-specific uptake and facilitating lysosomal escape 14. With the continuous innovations in synthetic technology, a variety of new functional polymers, either naturally derived or synthetic, have been used for vaccine delivery. Though the transfection efficiency of nucleic acids is significantly improved, the cytotoxicity and systemic toxicity still need to be addressed. Balancing the advantages and disadvantages of polymer carriers remains a pressing challenge. This section will introduce in detail the delivery strategies of nucleic acid vaccines using polymer carriers in recent years and discuss the influence of structural design on the delivery performance of carriers.

### 2.1. Types of polymers

#### 2.1.1 Polysaccharides

The natural origin of polysaccharides, such as chitosan, mannan, dextran, and beta-glucans, confers low toxicity, good biocompatibility and biodegradability, and immunoregulatory activity, are regarded as excellent vaccine carriers 15. Polysaccharides are composed of monosaccharides or disaccharides through glycosylic bonds. The presence of a large number of amino, carboxyl, and hydroxyl groups in the molecular structure is the basis of their extensive biological activities 16. Polysaccharide-based carriers can protect antigens from degradation, improve antigen delivery efficiency, and enhance immunogenicity 17. The positive charge of cationic polysaccharides encapsulates or binds to negatively charged nucleic acids, and the amino groups carried by polysaccharides help achieve the “proton sponge” effect for the cytoplasmic delivery of cargos.

**Figure 2:**
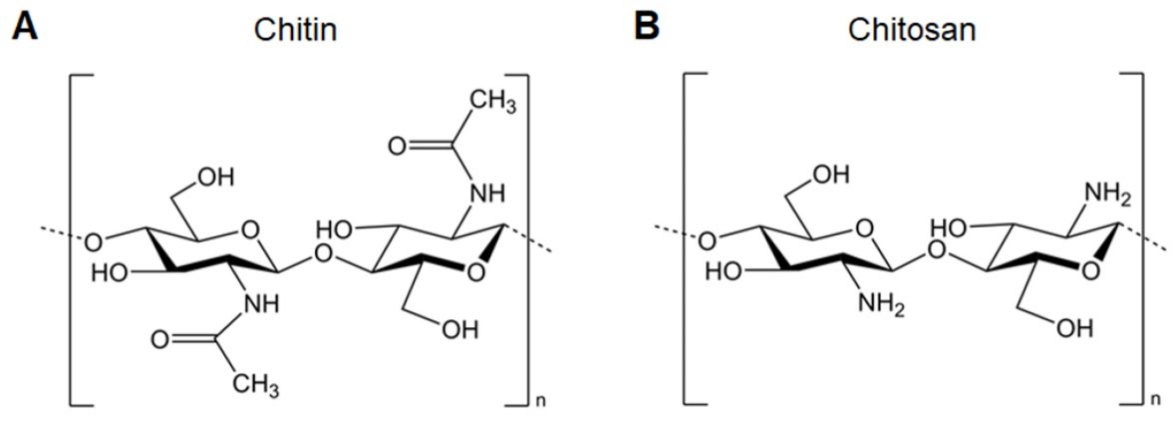
** A**. Chemical structure of chitin. **B**. Chemical structure of chitosan.

Chitosan, the most widely investigated and applied cationic polysaccharides for nucleic acid delivery, is composed of D-glucosamine and N-acetyl-D-glucosamine linked by β-(1,4) bonds. It is mainly derived from the deacetylation of chitin by removing more than 50% of the acetyl groups, converting them into amino groups (Figure 2). The high degree of deacetylation is beneficial for the electrostatic properties and solubility of chitosan, making it more suitable for loading nucleic acids and cellular uptake. Chitosan itself possesses immunomodulating properties which enhance antibody responses and cellular immunity post-vaccination via injection or mucosal routes 18. Turley et al. demonstrated that only chitosan with a high degree of deacetylation (>90%) enhances the generation of mitochondrial reactive oxygen species, leading to cGAS-STING and NLRP3 inflammasome activation of dendritic cells (DCs). These results reveal that the physicochemical properties of chitosan are important determinants for enhancing immune activation, paving the way for the design of vaccine adjuvants 19. In addition, low-molecular-weight chitosan shows improved solubility and enhanced intracellular nucleic acid release, which promotes downstream immune responses 20.

Furthermore, the cationic character of chitosan also allows for its interaction with mucosal surfaces, and chitosan can open tight junctions between epithelial cells transiently 21, making it suitable for triggering mucosal immunization. Intranasal or oral immunization with chitosan-/nucleic acid-based nano vaccines has been used against several pathogens, such as *Mycobacterium tuberculosis*, hepatitis B virus, *Schistosoma mansoni*, and *Streptococcus mutans* 22-26. Some approaches such as decoration with targeting moieties to APCs or incorporation of immunostimulatory molecules have also been investigated to further improve the efficacy of mucosal immunization 27.

#### 2.1.2 Poly(amino acid)s

There are 20 kinds of essential amino acids in biological systems, and more than 500 non-proteinogenic amino acids have been found. Amino acid-based polymers are considered promising biomaterials for biomedical applications 28. Due to the diversity of functional side groups in amino acids, a wide range of poly (amino acid)s (or polypeptides) with different chain lengths and compositions may be readily synthesized. The presence of amino acid moieties in the framework is beneficial to the solubility, biocompatibility, and biodegradability of amino acid-based polymers 29.

**Figure 3:**
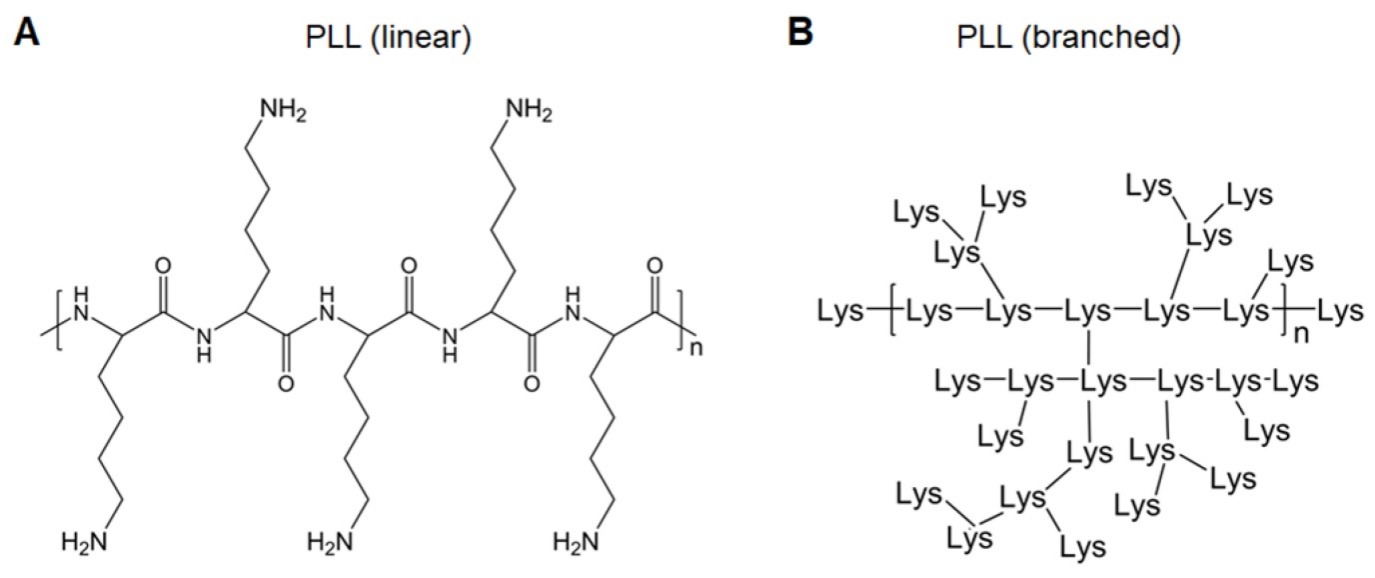
** A**. Chemical structure of linear PLL. **B**. Chemical structure of branched PLL.

Polylysines (PLLs), derived from lysines, are easily protonated and form complexes with negatively charged nucleic acids (Figure 3). Lysine-based cationic PLLs achieve high gene transfection efficacy with minimal cytotoxicity at low concentrations. Yu et al. constructed a polymer wire consisting of CpG motifs by hybridized chain reaction, followed by assembly with cationic PLLs to form nanospheres. This easily prepared polymer carrier enhanced the activation of immune cells through the continuous stimulation of lysosomal Toll-like receptor (TLR)9 of immune cells and further induced the death of cancer cells 30. Dendritic PLLs have better loading capacity and flexibility (Figure 3B) 31. Zhao et al. encapsulated the HA gene of H9N2 influenza virus plasmid DNA (pDNA) into dendrigraft poly-_L_-lysines (DGLs) via electrostatic interactions. After intramuscular injection, DGLs prevented pDNA degradation, assisted pDNA escape from endosomes, promoted antigen presentation, and induced effective cellular and humoral immune responses, thereby demonstrating that DGLs were good non-viral nucleic acid vaccine delivery carriers 32. To increase positive charge densities in the polymer structure, considerable attention has been paid to the development of highly branched poly(amino acid)s as gene delivery vectors 33, which will also show great potential in vaccine delivery.

#### 2.1.3 Polyamines

Natural polyamines, such as putrescine, spermine, and spermidine, play an important role in modulating gene expression, cell growth, and replication and translation of viruses. Polyamines are positively charged under physiological pH and ionic conditions, which form complexes with negatively charged nucleic acids, proteins, and phospholipids within cells 34. Owing to the highly positive charges of polyamines coupled with the properties of easy synthesis and effective polyplex formation with nucleic acids, they are considered important polycations for gene delivery. Synthetic polyamine analogs are capable of simultaneously targeting polyamine metabolism and the delivery of therapeutic nucleic acids, thereby displaying effective combinational therapy both in cancer and virus replication 35, 36.

**Figure 4:**
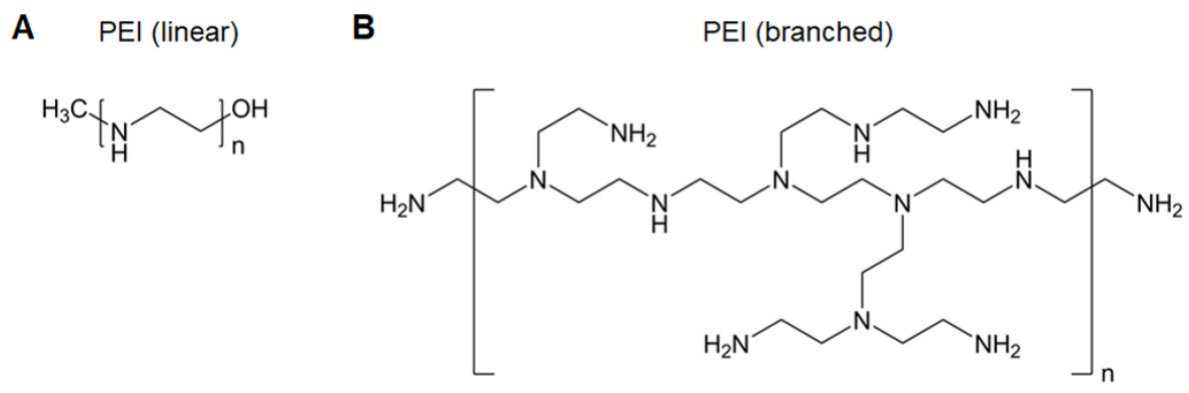
** A**. Chemical structure of linear PEI. **B**. Chemical structure of branched PEI.

Poly(ethyleneimine) (PEI), the most widely used polyamine for nucleic acid delivery due to its high charge density, is considered the “gold standard” in polymeric gene carriers (Figure 4) 37. The excellent “proton sponge” effect of PEI contributes to endolysosomal escape to avoid gene degradation and release into the cytoplasm. Nevertheless, the use of PEI is compromised by its high toxicity, as efficient gene transfer requires high-molecular-weight PEI that inevitably confers high cytotoxicity. Several strategies have been utilized to decrease the toxicity while maintaining the transfection efficacy of PEI, such as PEGylation to shield the positive charge, linking low-molecular-weight PEIs, and hydrophobic modification 38-41. We discovered that anchoring PEI at the surface of perfluorodecalin decreases PEI toxicity, which likely resulted from the decreased flexibility of PEI limiting its interactions with cell membranes and the oxygen dissolved in perfluorodecalin promoting cell growth 42.

In addition, PEI possesses immunostimulatory effects, such as stimulating DC activation, homing, and inducing cytokine production 43. This inherent immunostimulatory activity and its excellent transfection efficiency support PEI as a nucleic acid vaccine vector. Self-amplifying RNA (saRNA) enhances protein expression in comparison to mRNA, which is regarded as a next-generation nucleic acid vaccine. However, the large and complex RNA structure is difficult to translate in APCs, and saRNA also suffers from degradation 44. To address the challenges, Démoulins et al. investigated the effects of the molecular weight of PEI (2,500-250,000), saRNA: PEI ratio (weight: weight), and inclusion of (Arg)_9_ or TAT(57-57) cell-penetrating peptides on polyplex delivery. Two formulations [(saRNA/PEI_4,000_: 1:3) and (saRNA/PEI_40,000_: (1:2)/(Arg)_9_)] showed specific CD4^+^ and CD8^+^ T-cell immune responses in mice and pigs, which had a positive impact on the application of saRNA vaccines 45.

#### 2.1.4 Polyesters

Polyhydroxyalkanoate (PHA) represents biologically synthesized polyesters, which are naturally self-assembled inside bacteria. These PHA particles are core-shell structures composed of a hydrophobic core and outer proteins. These proteins may be used as an anchor for attaching antigens, thereby leading to the formation of antigen-coated PHA vaccines 46. More recently, chemically synthesized polyesters and their derivatives, such as poly (lactic-co-glycolic acid) (PLGA), poly (lactic acid), and polycaprolactone, have been employed in various biomedical applications, including tissue implants, tissue engineering scaffolds, drug delivery, and nucleic acid vaccine delivery. The distinctive feature of polyesters is their biodegradability because of the hydrolysis of ester bonds; therefore, several polyesters have been translated to clinical practice 47.

**Figure 5:**
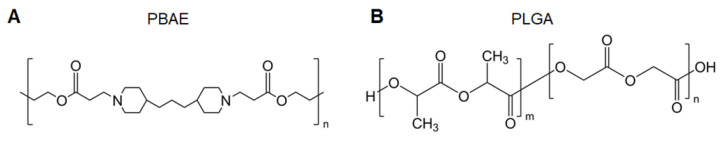
** A**. Chemical structure of PBAE. **B**. Chemical structure of PLGA.

Poly(β-amino ester) (PBAE) is a cationic polyester with the ability to form complexes with nucleic acids enhancing cellular uptake and the endolysosomal escape of polyplexes (Figure 5A) 48; PBAE polyplexes have immunostimulatory functions. Jewell et al. reported that PBAE particles induced the activation of DCs and macrophages 49, 50. A further study revealed that the molecular weight of PBAE had an important influence on immune activation. They synthesized a series of PBAEs with different molecular weights. Regardless of the initial molecular weight, the immunogenicity peak occurred when the molecular weight of hydrolyzed PBAEs decreased to 1.5-3 kDa 51. These results are beneficial to the design of biodegradable polymers to induce immune responses as vaccine carriers. DeMuth et al. prepared novel PBAE-based pDNA vaccine-loaded microneedle coatings using layer-by-layer techniques. The microneedle was applied to the skin, and the interstitial fluid led to rapid layer dissolution and the sustained release of in situ-formed PBAE/nucleic acid polyplexes, inducing 140-fold higher gene expression in primate skin than that associated with intradermal injections of naked DNA for eliciting robust immune responses against human immunodeficiency virus (HIV) antigens. The results indicated the great potential of this strategy for enhancing DNA vaccinations 52.

PLGA, a biodegradable copolymer composed of lactic acid and glycolic acid linked through ester bonds, is one of the most widely used polyesters in pharmaceutical sciences, medical devices, and other chemical fields (Figure 5B). PLGA-based microparticle-loaded DNA-encoding HIV antigens has entered a phase I clinical trial 53. However, payload release from traditional PLGA particles is usually time-consuming due to their slow degradation rate. Although the particles consist of low-molecular-weight PLGA, 13 days were needed to fully release the encapsulated DNA after APC uptake 54. Given this, Langer et al. developed a hybrid microparticle composed of degradable, pH-sensitive PBAE and PLGA as the carrier of genetic vaccines. The hybrid microparticle enhanced the intracellular delivery of DNA vaccines, producing 3-5 orders of magnitude higher transfection efficiency compared with that of PLGA formulations. Vaccination with this microparticle formulation in vivo led to robust antigen-specific immune responses 55. The combination of different polymers is not only simple but also provides the delivery system more functionality, whereas the proportion of different compositions is a potential point of concern. In addition, the combination of multiple materials will increase the structural complexity and may reduce the biocompatibility of the system, which needs to be considered when designing the combination.

#### 2.1.5 Polyamidoamines

**Figure 6:**
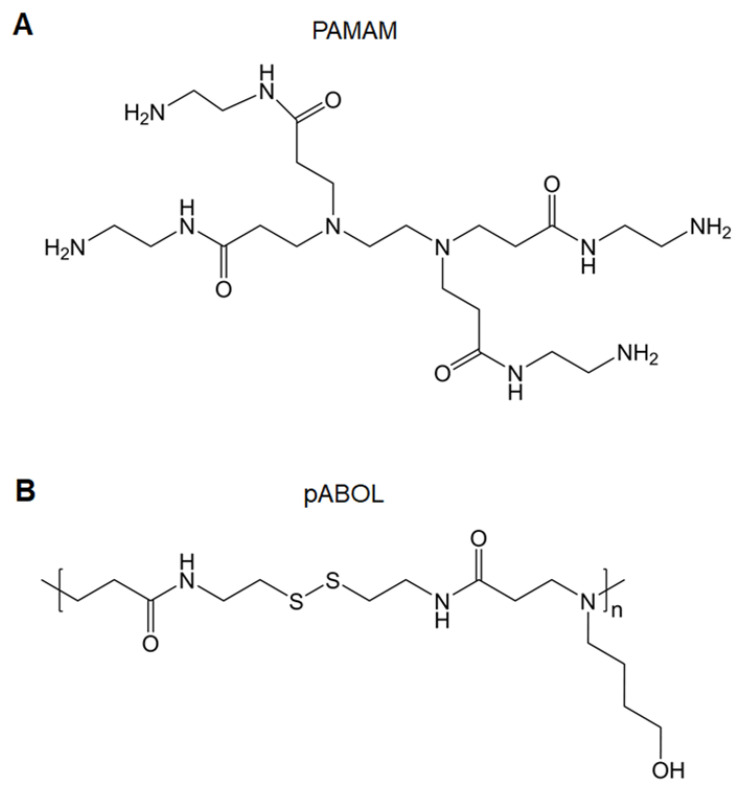
** A**. Chemical structure of PAMAM. **B**. Chemical structure of pABOL.

Polyamidoamines (PAMAMs) include tertiary amino and amido groups along their polymer chains. PAMAMs with a “tree-like” architecture are called PAMAM dendrimers, which are well-defined, highly branched, monodisperse, and homogeneous polymers (Figure 6A). There are three parts of PAMAM dendrimers: a core, repeating units, and shell functional group. Given its numerous amino groups and unique cavity structures, PAMAM dendrimers have been applied in drug and gene delivery 56. Other structures associated with PAMAMs are hyperbranched and linear polymers, which are structurally analogous to PAMAM dendrimers; these are also effective gene vectors. Unlike dendrimers, linear or hyperbranched polymers have better flexibility, facile synthesis, and improved gene complexation and transfection efficacies. In addition, the flexible structures offer polymers scope for modifications 57.

A functionalized amine-terminated surface may be modified with a variety of molecules that enables the adjustable physicochemical and biological properties of PAMAMs and their derivatives. For example, PEGylation improves hydrophilicity and shields the positive surface charges to reduce the toxicity of PAMAMs 58. Glycosylation (such as mannosylation, galactosylation, and lactosylation) or targeting peptide modification enhances cell-specific binding 59. Fluorination achieves excellent transfection efficacy and facilitates the self-assembly of PAMAMs 60. The co-delivery of immunoadjuvants (such as CpG) promotes immune-stimulating effects 61. To ameliorate the biocompatibility of PAMAMs, the synthesis of biodegradable dendrimers is an alternative strategy. Luckily, degraded PAMAM dendrimers are available on the market for gene transfection 62. At present, generation 4 PAMAM dendrimers are used for delivering Ebola DNA vaccines 63. Transcription-conjugated PAMAM dendrimer-loaded avian influenza virus DNA vaccines are used for transdermal vaccinations 64. Major histocompatibility complex (MHC)-II targeting peptide-conjugated PAMAM dendrimers are used for the delivery of tumor DNA vaccines 65. Linear poly(amido-butanol) (pABOL) (Figure 6B) has been synthesized to encapsulate influenza HA-encoding saRNA, showing that increasing the molecular weight of pABOL improves the saRNA delivery both in vitro and vivo 66.

### 2.2 Representative polymer-based formulations for nucleic acid vaccine delivery

The characteristics of multiple polymers used for nucleic acid delivery have been described above in detail, but the achievement of prophylactic and therapeutic purposes requires various pharmaceutical preparation technologies. Therefore, polymer-based vaccine formulations and their properties will be discussed in this section.

#### 2.2.1 Polymer particles

Particulate delivery systems are one of the most commonly used formulations. Particulate formulations are structurally similar to nano-sized viruses (e.g., severe acute respiratory syndrome coronavirus 2, influenza, and HIV) or micron-sized bacteria (e.g., *Staphylococcus aureus*, *Escherichia coli*, and *Salmonella*), which are pathogens that promote the evolution of the immune system. Since the immune system orchestrates in response to pathogenic particles, it is logical that similar sized and shaped particulate formulations trigger robust immune responses compared to those triggered by subunit or naked nucleic acid vaccines 67. It cannot be ignored that the development of NP-based tumor vaccines is in full swing, which utilizes NPs to prolong circulating time, control drug release, introduce tissue or cell targeting, enhance cellular uptake, facilitate endolysosomal escape, and improve transfection efficiency to induce potent cellular immune responses 68.

**Figure 7:**
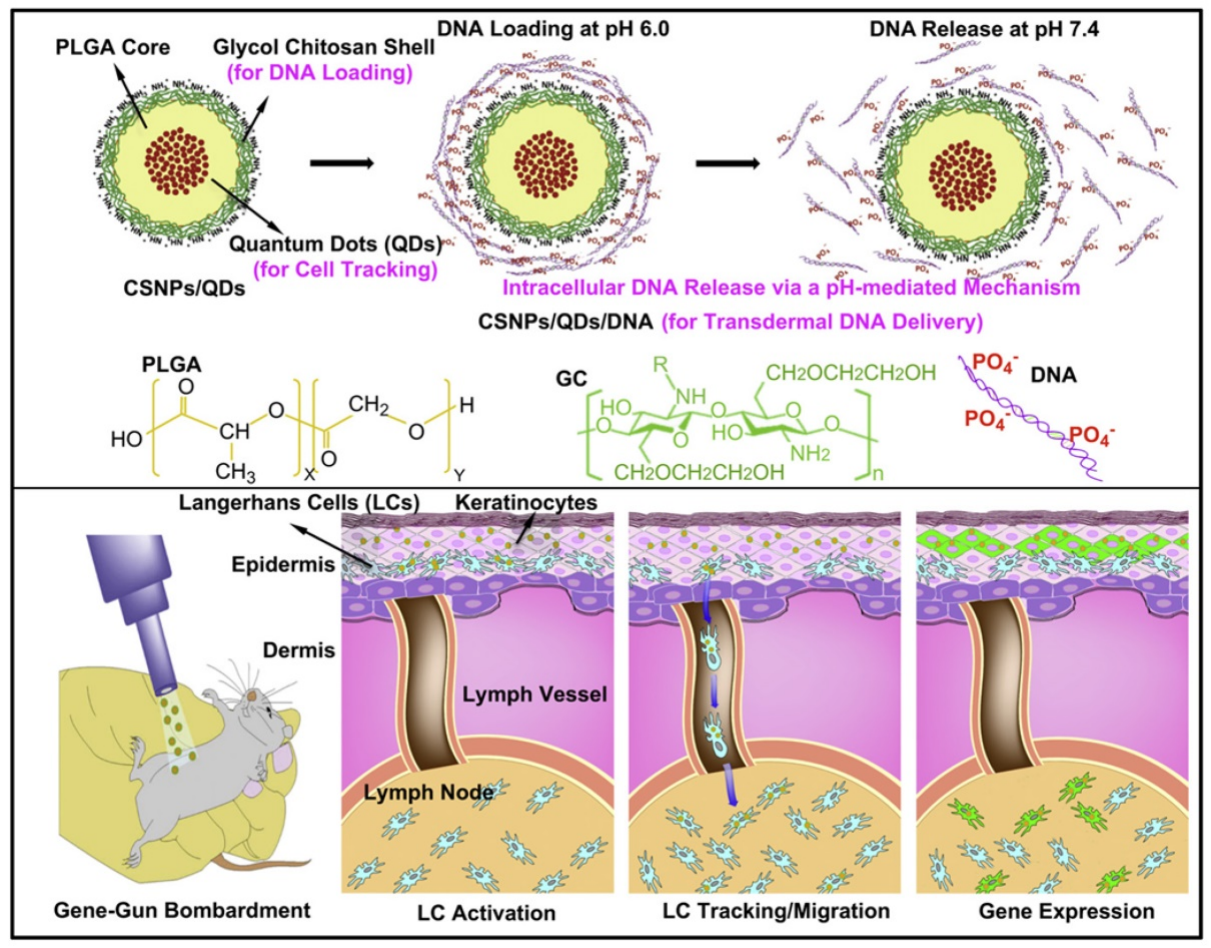
Schematic illustrations of the concept of multifunctional core-shell polymeric NPs: transdermal DNA delivery, tracking of Langerhans cell migration, a pH-mediated DNA release mechanism, and gene expression in LNs. Adapted with permission from 71, copyright 2010 Elsevier.

Particles composed of polymers and nucleic acids are formed mainly through physical encapsulation and electrostatic interactions. For example, PLGA alone has no positive charges, therefore, unmodified PLGA encapsulates nucleic acids using an emulsion technique, rather than electrostatic interactions. Immunization with these pDNA-containing PLGA microparticles achieved activated cytotoxic T lymphocyte (CTL) responses with a minimal DNA dose 69. However, the direct encapsulation of nucleic acids into particles always leads to large sizes and low encapsulation efficiencies. In addition, for PLGA particles, encapsulated nucleic acids may suffer from degradation as PLGA hydrolysis, resulting in low transfection efficiency. To address this issue, PLGA particles are modified with cationic features to condense nucleic acids through electrostatic interactions 7. Studies have involved chitosan, PEI, or dendrimers being mixed with PLGA 70-72, cetyl trimethylammonium bromide being absorbed onto PLGA particles 73, and PEI being covalently conjugated to the surface to create cationically modified PLGA particles 74. Lee et al. developed an NP composed of a PLGA core and positively charged glycol chitosan shell, wherein quantum dots were loaded in the core of PLGA NPs to track Langerhans cell migration, and nucleic acids were absorbed on the positively charged shell (Figure 7). pH-responsive DNA release and effective gene expression were detected in lymph nodes (LNs). NPs have shown great potential in monitoring the immune system and vaccine development 70. In addition to modified PLGA particles, the application of cationic polymers for gene vaccine delivery should be a more straightforward method. Chahal et al. developed dendrimer-RNA NPs that protect against a broad spectrum of pathogen challenges, such as Ebola virus, influenza virus, and *Toxoplasma gondii* with only a single dose. In contrast to cell culture or fertilized egg systems, the preparation of this vaccine system requires approximately 1 week. Therefore, this technology may be used to develop rapid-response vaccines against sudden outbreaks and evolving pathogens 75.

Present evidence indicates that the size of particulate vaccines is closely associated with their immune effects (Table 1). First, the size of particulate vaccines determines the antigen transport route to the LN after peripheral injection. Particles with a diameter less than 10 nm directly enter the blood circulation through the capillaries, while particles 10-200 nm in size traffic into lymphatic capillaries 76. Other large particles (>200 nm) are phagocytosed by peripheral DCs and transported to LNs 77. There is an abundance of APCs, T cells, and B cells residing in LNs; therefore, antigens reaching the LNs trigger stronger immune responses than those transported by peripheral DCs 76, 78. Second, DC uptake is also influenced by the particle size. Akashi et al. has extensively studied poly (γ-glutamic acid). They prepared a series of different sizes (30-200 nm) of poly(amino acid) NPs and found that the amount of intracellular NPs increased with increasing size in bone marrow-derived dendritic cells (BMDCs) and RAW264 cells in vitro. The in vivo DC uptake showed that the amount of large NPs taken up by a single DC was higher than that of small NPs. However, the number of positive DCs showed inverse results, which might be due to the different routes to LNs between different sizes of NPs, i.e., the small NPs migrated to LNs through direct drainage faster than the large ones via two steps of local DC uptake and transportation 79. Third, the DC activation correlates with the sizes of particles. Akashi et al. discovered that the induction of the DC maturation potential increased as the size of polymer particles decreased. The interaction between larger total surface areas of small particles and the surfaces of DCs contributes to the effectiveness in DC maturation 79. Fourth, the size of particles plays an important role in intracellular localization. Following APC uptake, particles will reach endolysosomes. For nucleic acid vaccines, the particles carrying nucleic acid vaccines should potentially escape from endolysosomes. Small particles appear to be more efficient in endolysosomal escape, thereby promoting cytoplasm localization, whereas larger particles are retained in the endolysosomes 80. Finally, sufficient retention time in LNs confirms the interactions with APCs. Studies have revealed that large particles show good retention in LNs 81. Therefore, rational designs of vaccine particle size will achieve orchestrated vaccine potency.

In addition to the particle size, the surface charge shows some relationships with the immunogenicity of vaccines (Table 1). The positive charge is an essential parameter of polymeric carriers for gene delivery, showing improved internalization by APCs than those with a negative or neutral charge 82. Cationic polymers are considered to promote endolysosomal escape through the “proton sponge” effect, which is important for nucleic acid delivery. Wu et al. revealed that DC activation is driven by cationic nano vaccines by promoting the secretion of tumor necrosis factor-α (TNF-α) and IL-12p70, which is more efficient than that of anionic nano vaccines 83. Both positively and negatively charged particles induce immune responses; however, cationic nano vaccines may induce a more pronounced Th1 immune response than that induced by anionic nano vaccines, which offer the potential for fighting against cancer or intracellular infections 83. It should also be noted that many negatively charged glycosaminoglycans and proteins exist in the tissue interstitium and fluid; therefore, positively charged particles may adhere and fail to drain into lymphoid tissues, while negatively or neutral-charged particles avoid being trapped 78. Therefore, there should be a balance between the different charges when particles are in different environments.

Apart from size and charge, the rigidity and shape of polymeric particles also affect their immune effects (Table 1). In general, rigid particles are more likely to be taken up by APCs, whereas flexible particle uptake tends to consume more energy, resulting in slower uptake. Cui et al. prepared mechanically tunable polypeptide particles composed of poly(_L_-glutamic acid) cross-linked to CpG. The rigidity of the particles was changed by altering the cross-linker concentration. They observed that DC association and activation occurred in a rigidity-dependent manner 84. The study implied that the mechanical properties of particles have an important influence on immunity. Regarding the shape of particles, the rod-shaped polymeric particles were more efficient at cellular uptake than spherical particles 85. In addition, the angle between cells and rod-shaped particles was important in uptake. The tangential angle is not conducive for uptake due to the high associated energy expenditure 86.

In addition to the aforementioned polyplexes or core-shell particles, other particles, such as polymer micelles, lipopolyplexes, and polymer-engineered inorganic NPs, have also been investigated for gene vaccine delivery. For example, Layek et al. modified chitosan with hydrophobic L-phenylalanine and α-D-mannopyranosylphenyl isothiocyanate for APC targeting. The obtained cationic polymer micelles formed a complex with DNA vaccines, triggering potent cellular immune responses in vivo 87. The lipopolyplex combined the advantages of both polymer and lipid systems 88. Shen et al. developed a vector consisting of a PBAE/mRNA core encapsulated into a 1,2-dioleoyl-*sn*-glycero-3-ethylphosphocholine/1,2-dioleoyl-*sn*-glycero3-phosphatidyl-ethanolamine/1,2-distearoyl-*sn*-glycero-3-phosphoethanolamine-*N*-[amino(polyethylene glycol)-2000 lipid shell 89. The liposome shell effectively protected the internal mRNA from degradation and reduced the interaction between mRNA and non-APCs, providing an enhanced effect on DC internalization than that by polymer carriers. Xu et al. prepared poly (diallydimethylammonium chloride) - and PEI-coated Au nanorods, which significantly enhanced APC activation and cellular and humoral immunity compared to naked pDNA 90.

Considering the flexibility of polymer composition and controllable fundamental parameters, polymeric particles are widely used for gene vaccine delivery. As the properties of particles required in vivo are dependent on a particular application, the facile modification of polymers and their stimulus-responsiveness will help vaccine design. In addition, similar to that of lipopolyplexes or polymer-engineered inorganic NPs, the integration of different technologies may be one of the prospective areas for nucleic acid vaccine delivery.

#### 2.2.2 Polymer hydrogels

Hydrogels are hydrophilic macro-scaled gel systems with a three-dimensional network structure, high water content, controllable drug release rate, and good biocompatibility, which create a new area for the delivery of various therapeutic agents, including nucleic acid vaccines 91. The notable capacity of hydrogels ensures concurrent encapsulated vaccines, adjuvants, cytokines, or nanostructures. In comparison with NPs, macro-scaled hydrogels are commonly applied at the topical site for the slow continual release of cargos or controlled release upon physiological or external stimuli 91. Natural polysaccharides and synthetic polymers have been used to prepare hydrogels for vaccine applications 92, 93.

**Figure 8:**
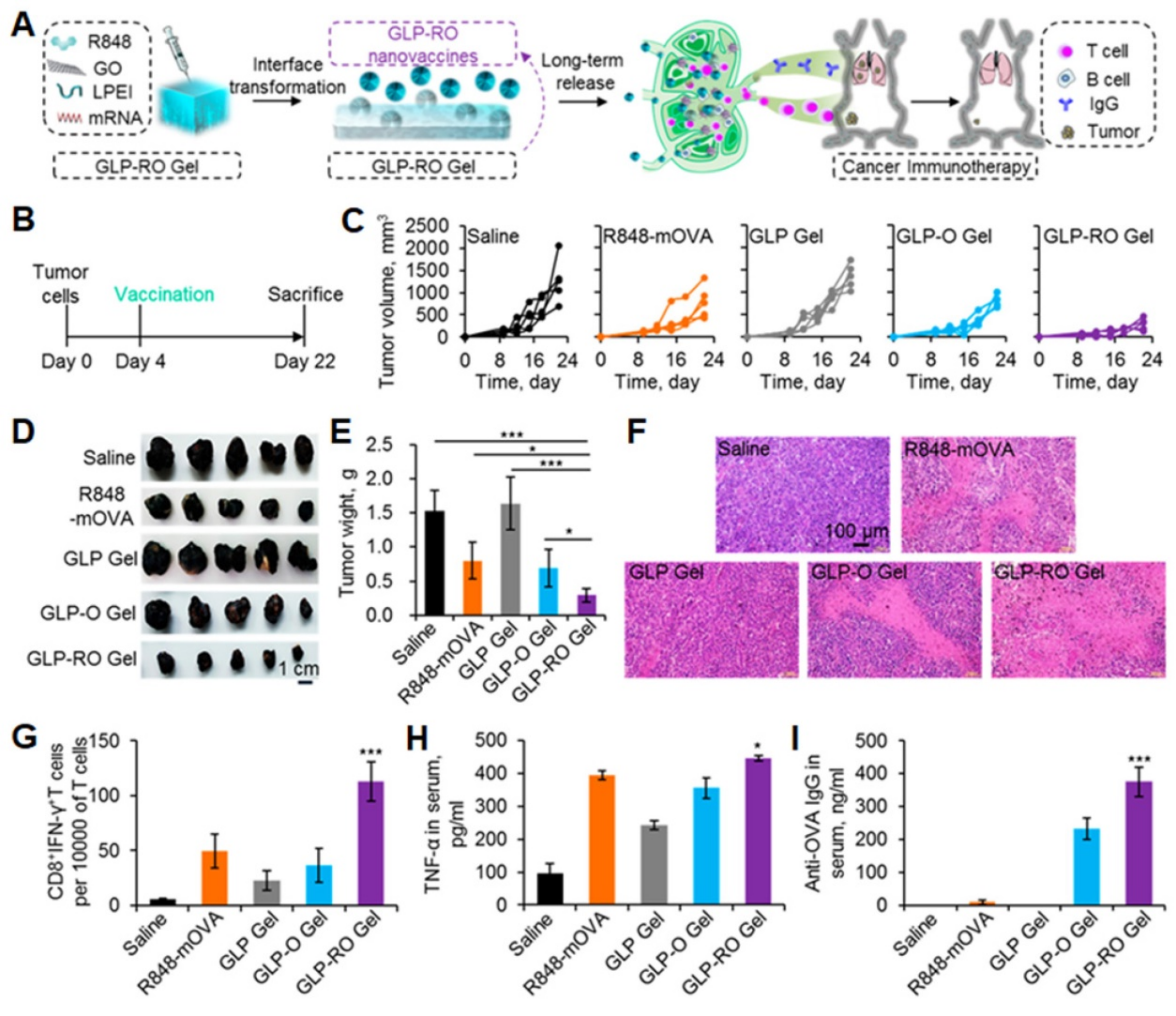
** A**. GO and low molecular weight PEI (LPEI) are fabricated to form the injectable hydrogel (GLP-RO Gel) to encapsulate mRNA and R848. **B**. Illustration of the treatment intervals. **C**. Growth curves, **D**. gross images, and **E**. weight of tumors. **F**. H&E images of tumor tissues. **G**. Flow cytometry analysis of T cells in splenocytes. **H**. ELISA analysis of TNF-α and **I**. OVA-specific IgG in serum. Adapted with permission from 99, copyright 2021 American Chemical Society.

Thermo-sensitive hydrogels that perceive physiological temperature and transform into gelation endow the delivery system with convenient spatial and temporal application and release control 94. To achieve sustainable vaccine release, Bansal et al. first prepared pDNA-loaded PLGA-chitosan NPs and then dispersed the NPs into poloxamer 407 gel, which was in a liquid state at 4 °C and transformed into a soft gel at physiological temperature (37 °C). They characterized the physicochemical properties and evaluated the vaccine potential both in vitro and in vivo. The hydrogel showed promises for future rabies prevention and immunocontraception 93. The Lee and Jeong group have extensively studied a temperature-sensitive copolymer: poly (ε-caprolactone-*co*-lactide)-*b*-poly (ethylene glycol)-*b*-poly (ε-caprolactone-*co*-lactide) (PCLA). They prepared an in situ-forming injectable hybrid hydrogel by conjugating PCLA with bovine serum albumin. DNA vaccine polyplexes were loaded through ionic interactions. After injections, the hybrid hydrogel spontaneously assembled into a microporous hydrogel depot, allowing for the recruitment of DCs 95. Furthermore, to strengthen the stability of the hydrogel during implantation, they conjugated adhesive dopamine and PCLA to hyaluronic acid (HA-PCLA). pDNA polyplexes for expressing antigens and granulocyte-macrophage colony-stimulating factor (GM-CSF) for recruiting immune cells were coloaded in the microporous network of HA-PCLA hydrogel. The amount of recruited DCs was 6-fold higher than that of the conventional PCLA counterpart, suggesting that the hydrogel possesses the potential for disease immunotherapy 96. Recently, organic-inorganic hybrid materials have the potential to combine the physical and chemical properties of each component synergistically toward an enhanced vaccine delivery 97. Graphene oxide displays various immune effects to improve antigen immunogenicity by promoting cytokine secretion, DC recruitment, and antigen depot 98. Yin et al. reported an injectable in situ transformable hydrogel composed of graphene oxide and PEI that was formed via electrostatic interactions (Figure 8). R848 and ovalbumin-encoding mRNA were encapsulated into the hydrogel through π-π stacking and electrostatic interactions. After injection into subcutaneous layers, the hybrid hydrogel converted into NPs gradually. The released NPs deliver R848 and mRNA to LNs, thereby translating ovalbumin antigens for durable and efficient cancer therapy 99.

#### 2.2.3 Polymer microneedles

**Figure 9:**
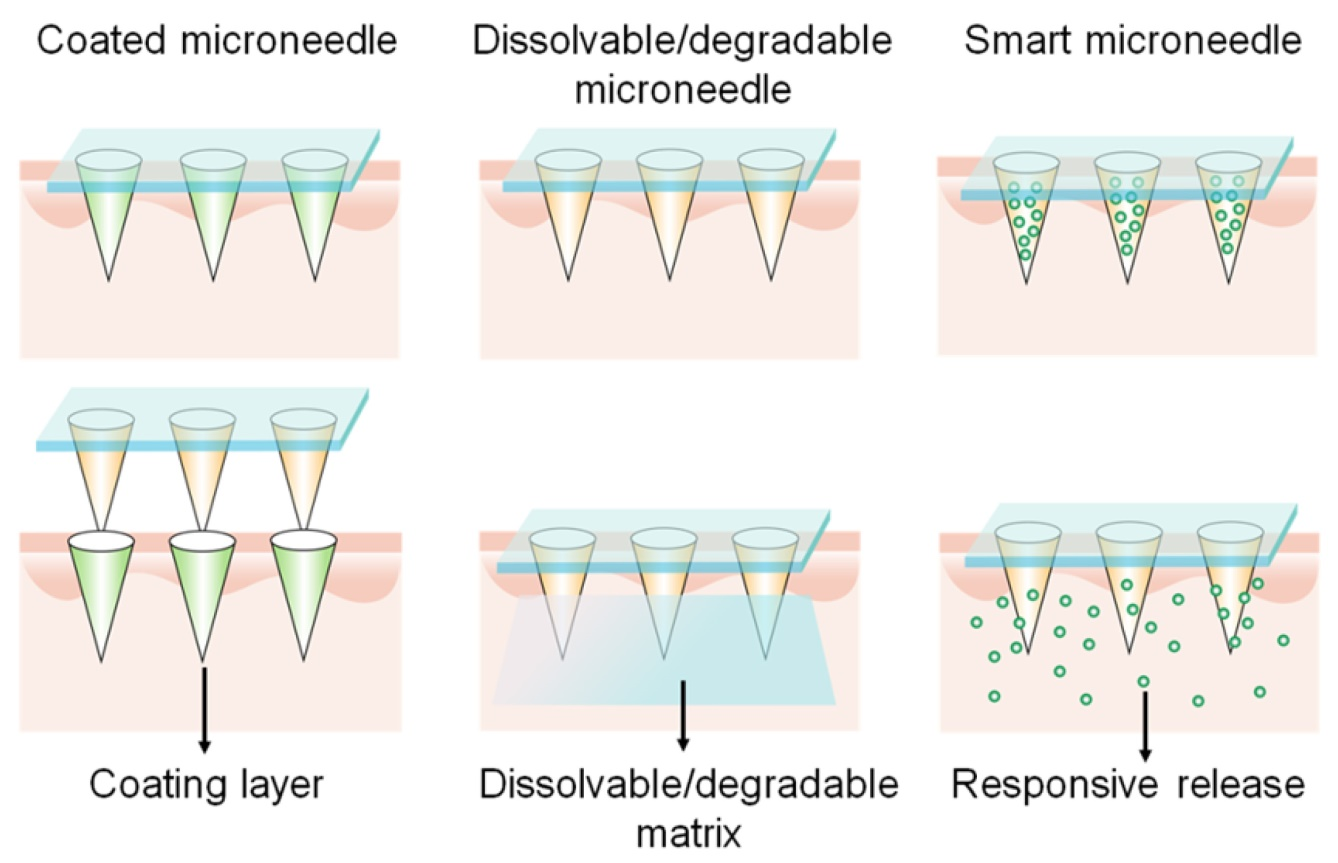
Representative microneedles including coated, dissolvable/degradable, and smart microneedles.

The skin is an ideal site for immunization due to its abundant APCs presence. However, it is difficult to achieve precise delivery to the intradermal layer using a needle and syringe without a medical professional. In the past decade, transdermal vaccinations using microneedle arrays have become a booming field for investigations. Microneedle patches represent a safe, pain-free, patient-compliant, and self-administered transcutaneous delivery system 100. Nowadays, this advanced technology is used in gene vaccine delivery percutaneously to produce effective vaccinations. A microneedle array, typically dissoluble/degradable, or removable after application, should have the properties of sufficient mechanical strength to traverse the stratum corneum and reach the epidermis, with subsequent efficient release of the cargos 101. There are several representative microneedles, such as coated, dissolvable/degradable, and smart microneedles (Figure 9). Coated microneedles are composed of solid microneedles and coated drug payloads. Although coated microneedles have sufficient mechanical force, they have limited surface area for drug loading and need to dispose of biohazardous microneedles after usage, and thus are less commonly investigated than dissolvable/degradable, and smart microneedles 102.

Dissolvable or degradable microneedles refer to the utilization of soluble or degradable matrix materials to prepare microneedles which dissolve or degrade to release cargos after insertion into the skin. The release rate and therapeutic effects are closely related to the dissolution kinetics of the matrix. Chitosan, polyvinyl alcohol (PVA), and polyvinyl pyrrolidone (PVP) are representative dissolvable matrices, while PLGA is a typical degradable material for preparing microneedles 102. Yang et al. prepared an Ebola virus pDNA vaccine microneedle containing two components: PLGA-PLL/poly-γ-glutamic acid NP-loaded DNA and PVA/PVP matrix materials. PVA and PVP endowed the microneedles with the mechanical force for insertion into the skin with the ability of rapid dissolution. Negatively charged poly-γ-glutamic acid was used to weaken the NP structure to increase DNA release. The microneedle patch induced a strong immune response post-immunization in vivo 103. McCarthy et al. has extensively studied dissolvable microneedles for DNA vaccine transdermal delivery. They discovered that the application of lyophilization increased the loading of pDNA NPs within PVA microneedles 104. They also used different dissolvable polymers [PVP (*M*_w_ = 58,000 Da), PVP (*M*_w_ = 360,000 Da), PVA (*M*_w_ = 13-23,000 Da), PVA (*M*_w_ = 9-10,000 Da)] to construct microneedles and compared the mechanical properties and DNA polyplex loading capacities. The results demonstrated that DNA was degraded when loaded in PVP, whereas PVA arrays weren't, resulting in a 10-fold higher transfection efficiency than that of PVP arrays. This study suggests that PVA-based microneedles are suitable for nucleic acid delivery 105.

**Figure 10:**
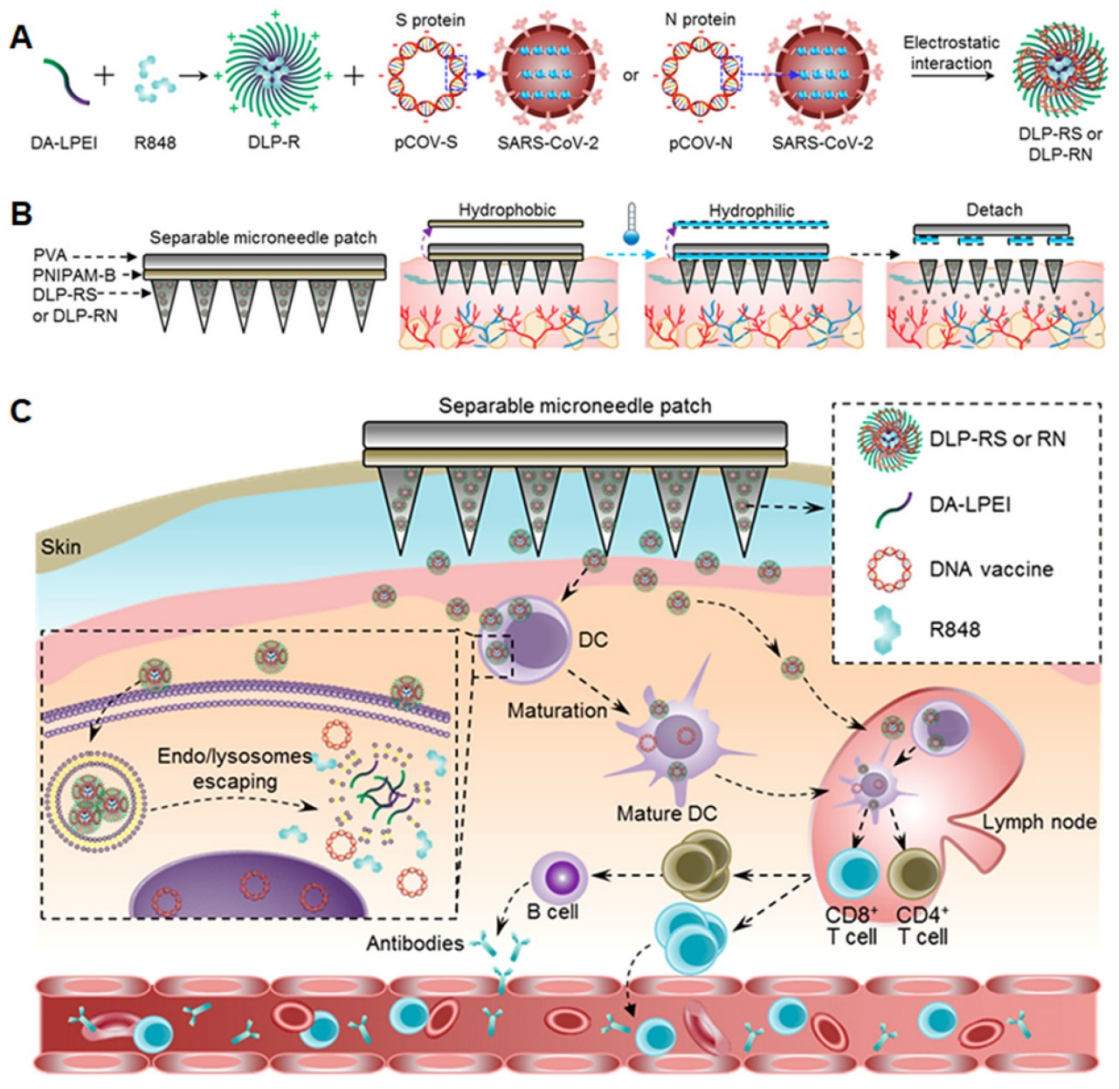
** A**. Deoxycholic acid conjugated LPEI (DA-LPEI) was applied to encapsulate R848 and S- or N-protein encoding DNA vaccines (DLP-RS or RN). **B**. The backing layer of microneedles can be separated from the skin and leave the microneedles in the skin by controlling temperature. **C**. Physiological mechanism of separable microneedle patch mediated antiviral immunity. Adapted with permission from 109, copyright 2021 American Chemical Society.

Smart microneedles may be triggered to release cargos in response to endogenous or exogenous signals. This technology provides opportunities for on-demand release in a controllable manner. For example, the Jeong and Lee groups designed a series of pH-responsive microneedles for DNA vaccine delivery. Heparin and albumin were used for constructing the pH-responsive microneedle via layer-by-layer deposition. The albumin exhibited a positive charge when the environmental pH value is 4.9 and transformed to a negative charge in physiological pH conditions. Therefore, the microneedles responsively released the loaded polyplex post-insertion into the skin. The released mannose-modified polyplexes then targeted APCs and delivered DNA vaccines 106. Furthermore, they also prepared ultra-pH-responsive microneedles using oligo(sulfamethazine)-*b*-poly(ethylene glycol)-*b*-poly(amino urethane) to deliver DNA polyplexes and immunoadjuvants 107, 108. The copolymer was positively charged at pH 4.03 and became negatively charged when exposed to physiological pH values. In addition, to achieve rapid separation of the backing layer from the microneedle, a separable microneedle patch to deliver DNA vaccines to fight against COVID-19 was developed (Figure 10) 109. First, deoxycholic acid was conjugated to PEI to simultaneously encapsulate adjuvant R848 and antigen-encoding DNA. PVA was then used to form the microneedle patch to penetrate the epidermis, wherein a separating layer consists of poly (*N*-isopropylacrylamide-*co*-butyl acrylate) (PNIPAM-B), a thermally responsive polymer that was hydrophobic at room temperature and became soluble at temperatures below 14 °C. Thus, the backing layer could be removed from the skin by controlling the skin temperature for several minutes. This thermal-responsive technology makes microneedle patches more available and accessible against infections or severe diseases.

## 3. Challenges relevant to polymer-based nucleic acid vaccine delivery

### 3.1 Problems associated with nucleic acid vaccines

Vaccinations are the most effective tool for preventing the spread of various infectious diseases worldwide. The World Health Organization reported that 2-3 million individuals are protected from death by vaccinations annually. Although vaccines exert great potential in the prevention of multiple infections, they still face significant challenges for the treatment of emerging and several major diseases, such as COVID-19, cancer, and HIV infection 110. Limitations in traditional vaccines and the severity of global diseases prompt the development of novel vaccines, including nucleic acid vaccines, which have garnered great attention in the prevention and treatment of diseases 111. Unfortunately, few nucleic acid vaccines have been licensed for human use. The reasons underlying the unsatisfactory translation rate may be summarized as follows: (1) the potential safety risks for human use and (2) modest immune responses elicited by nucleic acid vaccines in humans fail to exert desired outcomes for disease treatment.

The safety of vaccines is a prerequisite for their clinical application. For instance, DNA vaccines may integrate into the genome of host cells, which may cause the activation of proto-oncogenes and thus induce cancer 112. However, the probability of this occurrence is relatively low, but is still a necessary evaluation index for vaccines undergoing clinical trials 113. In addition to the potential safety risks, the weak immune response induced by DNA vaccines in clinical trials is another critical factor that hinders their clinical translations. In the 1990s, studies demonstrated that DNA vaccines successfully produced a strong immune response against the influenza virus in mice 114. However, more recent studies have shown that the immune effect generated by DNA vaccines in large animals or humans is limited, due to the diverse physiological barriers, such as insufficient cellular uptake, and the nucleic acid degradation by endogenous nuclease 111. For RNA vaccines, except for delivery-related obstacles, its vulnerability and inherent instability also limit their immune effects and industrial production. Additionally, most naked nucleic acids injected accumulate in non-target tissues, causing undesired immune activation via binding to TLRs 110, 111. Collectively, an exquisite delivery vector must be designed to optimize the delivery efficiency and immune effects of nucleic acid vaccines.

### 3.2 Problems with polymeric delivery

Polymeric materials have been widely studied for nucleic acid vaccine delivery, which protect the payload from degradation and endow it with improved transfection efficacy. There are a variety of polymeric materials with excellent biocompatibility, among which PLGA has been approved by the U.S. FDA for various medical applications 115. However, polymers for nucleic acid delivery still face many challenges that need to be resolved. First, APCs are key immune cells responsible for bridging the innate and adaptive immune systems. They are indispensable immune cells for the initiation of adaptive immune responses. Therefore, efficiently delivering antigens to APCs is critical for immune activation. However, the efficiency of APC-targeted antigen delivery by polymers alone is currently poor, thereby resulting in weak immune responses 116. Second, most polymers used for nucleic acid delivery are cationic polymers with inevitable cyto- and systemic toxicities 117. For example, PEI is a cationic material widely used in nucleic acid delivery based on its excellent cellular entry performance and pH buffering capacity 118. However, PEI with high molecular weight has significant physiological toxicity, which hinders its widespread application. Third, cationic polymers may be trapped in the negatively charged extracellular matrices before they deliver the nucleic acid to APCs, thus severely reducing the delivery efficiency and immune activation of nucleic acid vaccines 76. Fourth, inadequate dissociation of polymer vectors and subsequently limited exposure of nucleic acid vaccines will significantly affect the transfection efficiency of nucleic acid vaccines, thereby resulting in a poor immune response 119. Therefore, there remains plenty of scope to improve the performance of polymeric materials for nucleic acid vaccine delivery.

### 3.3 Physiological barriers for nucleic acid vaccine delivery in vivo

Nucleic acid vaccines are less prone to degradation by nuclease using polymer vectors, but their immune response remains hindered by the multiple physiological barriers during in vivo delivery 111. In order to improve the immune effect of nucleic acid vaccines, different administration routes (such as intravenous, oral, intradermal, subcutaneous, and intranasal) have been examined to study their influence on the immune effect of nucleic acid vaccines 120. The immune activation is enhanced by directly delivering nucleic acid vaccines to lymphatic organs/tissue. Nucleic acid vaccines delivered via distinctive immune routes will encounter different physiological barriers and eventually reach different lymphatic systems 7. The physiological barriers for nucleic acid vaccine in vivo delivery fall into three categories: (1) at the organism level, (2) at the organs/tissues level, and (3) at the cellular level (Table 2).

#### 3.3.1 Physiological barriers for nucleic acid vaccine delivery at the organism level

Vaccine delivery to systemic circulation via intravenous administration will inevitably encounter systemic biological barriers. For example, protein adsorption in serum significantly increases the particle size and hinders the effectiveness of the carriers. The formation of a “protein corona” around particles will alter their surface properties, induce aggregation, and finally reduce vaccine efficacy 121. In salt environments, the cationic polymer-formed particles face the challenge of colloidal stability as well. The nucleic acid will suffer from the degradation by DNase and RNase enzymes in serum. In addition, the rapid clearance rate in circulation is another major barrier when administered intravenously. The delivery systems must have the ability to escape from the reticuloendothelial system that would otherwise remove the formulations from the body. Moreover, off-target effects require a high dose to induce an adequate immune response, which may be accompanied by side effects 122.

#### 3.3.2 Physiological barriers for nucleic acid vaccine delivery at the organ/tissue level

##### (1) Physiological barriers for nucleic acid vaccines delivered to LNs

For nucleic acid vaccines, efficient delivery to LNs is critical to obtain desired immune effects since the LNs contain diverse immune cells, including a large number of APCs 123. Additionally, nucleic acid vaccines may be delivered to LNs through intradermal, subcutaneous, and intramuscular injections, but all of them will encounter some physiological barriers before reaching the destination. For instance, nucleic acid vaccines may be taken up by cells at the injection site (such as dermal fibroblasts and myocytes), and subsequently, the encoded protein antigens are released and captured by DCs at the injection site and drained to LNs, facilitating the generation of a T cell immune response 7. Alternatively, nucleic acid vaccines may also be directly taken up by DCs at the injection site, which may result in specific immune tolerance responses against encoded protein because DCs have not yet reached the appropriate site for antigen presentation 76. Furthermore, the LN-targeting ability of polymer carriers without specific targeting ligands modification is limited. Cationic polymer vectors will be trapped within the extracellular matrix composed of negatively charged polysaccharides and proteins before they arrive at the target tissue, which severely impacts their immune effects 76.

##### (2) Physiological barriers for nucleic acid vaccines delivered to the spleen

The spleen is the largest secondary lymphoid organ in the human body, which performs diverse immunological functions 124. Consequently, the spleen is also a reasonable target for nucleic acid vaccines to induce desired immune responses via intravenous injection. Nevertheless, as discussed in 3.3.1, cationic polymer carriers interact with proteins during in vivo delivery, thus causing the dissociation or aggregation of delivery systems 7. Additionally, nucleic acid delivery carriers may be delivered to non-target organs (such as the lungs) due to the existence of the lung shearing force, limiting their further immune induction 125. Moreover, the innate immune cells in the blood may directly take up the nucleic acid vaccines, resulting in the rapid elimination of nucleic acid vaccines or the activation of innate immunity and even the formation of inflammation 7, 125. Collectively, nucleic acid vaccines will encounter various delivery barriers in circulation, which significantly impacts their spleen-targeted efficiency.

##### (3) Physiological barriers for nucleic acid vaccines delivered to mucosa-associated lymphoid tissues

Mucosa-associated lymphoid tissues are a lymphatic system distributed throughout the body, which include nasal- and gut-associated lymphoid tissues, among others 126. Notably, the mucosa-associated lymphoid tissue is a suitable target for nucleic acid vaccines to enhance mucosal immunity. Nucleic acid vaccines can be delivered to nasal-associated lymphoid tissue through intranasal immunization, encountering diverse physiological barriers through the process 7. For instance, nucleic acid vaccines must penetrate the mucosa and undergo epithelial or microfold cells (specialized epithelial cells)-mediated transport, before reaching the nasal-associated lymphoid tissue, but the mucus secreted by mucosa may rapidly eliminate the nucleic acid vaccine, which is not conducive for nucleic acid vaccines to passively transport to nasal-associated lymphoid tissue and the eventual induction of mucosal immunity 7. Additionally, nucleic acid vaccines may be delivered to gut-associated lymphoid tissues through a commonly used oral route; however, they will suffer from severe physiological conditions, for instance, the extremely acidic environment in the stomach, abundant intestinal microbes, and nuclease that lead to degradation 7, 127.

In brief, nucleic acid vaccines administered via different routes will encounter distinctive extracellular physiological barriers before reaching the corresponding lymphatic organs/tissues, which severely limit their immune effects.

#### 3.3.3 Physiological barriers for nucleic acid vaccine delivery at the cellular level

To induce strong immunity after reaching lymphatic systems efficiently, it's also necessary for nucleic acid vaccines to overcome a series of intracellular physiological barriers. The cell membrane is the first physiological barrier for nucleic acid vaccines at the cellular level, and the cellular uptake efficiency of nucleic acid vaccines alone is poor due to its negative charges and inherent hydrophilic nature 128, 129. Although various polymer vectors have been used for nucleic acid vaccine delivery, their cell uptake efficiency remains limited 128. Furthermore, upon reaching the intracellular region, nucleic acid vaccines must rapidly complete lysosomal escape to avoid nuclease degradation in the endolysosomes, and the pH of late lysosomes may be as low as 4.5, which may affect the nucleic acid stability 130. Additionally, dissociation of the delivery platform and the subsequent exposure of nucleic acids are critical for the downstream expression processes of nucleic acid vaccines. However, the strong electrostatic interactions between cationic polymer vectors and payload nucleic acid vaccines make it difficult to completely dissociate the delivery complex, leading to poor transfection efficiency 119. Finally, mRNA vaccines must be delivered to the cytoplasm, whereas DNA vaccines must enter the nucleus via a more complicated process. In order to prevent naked DNA from being degraded by nucleases in the cytoplasm 111, DNA vaccines must enter the nucleus quickly through nuclear pore complexes (NPCs). Proteins with a size of less than 9 nm or molecular weight of less than 40 kDa may passively diffuse into the nucleus through NPCs 131, but larger nucleic acid vaccines (greater than 2000 base-pairs) fail to achieve nuclear transport 7.

## 4. Strategies to enhance polymer-based nucleic acid vaccine delivery

**Figure 11:**
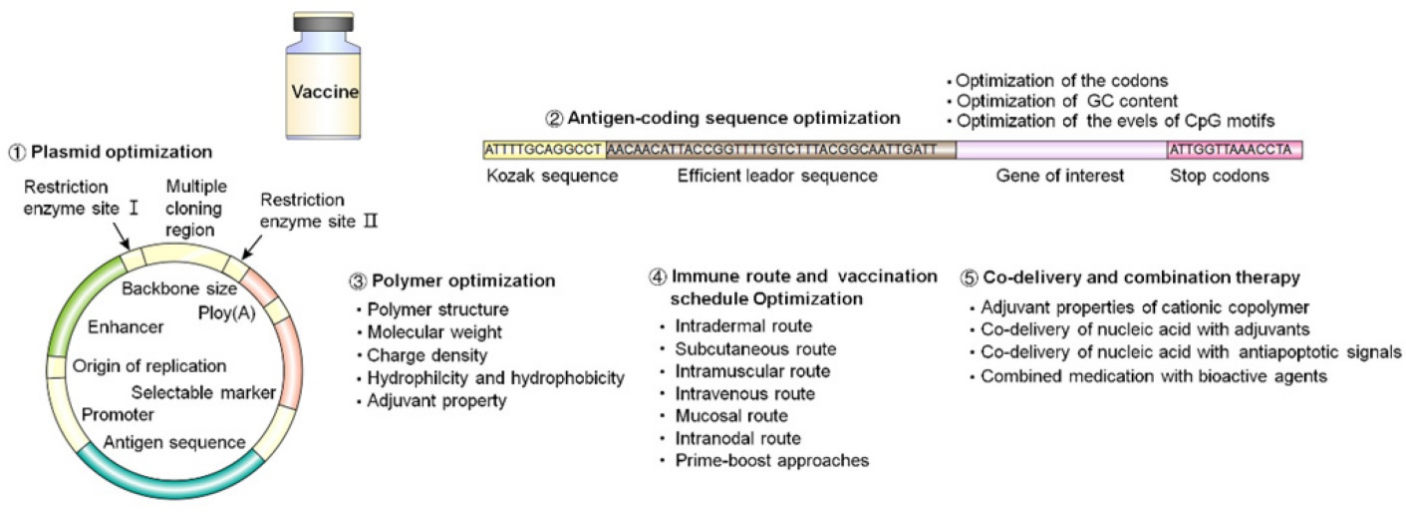
Summary of strategies for enhancing the efficacy of nucleic acid vaccines.

Multiple strategies to improve the design and efficacy of nucleic acid vaccines include plasmid optimization, antigen-coding sequence optimization, polymer optimization, immune route and vaccination schedule optimization, and the use of co-delivery and combination therapy (Figure 11). In this article, we mainly discussed the optimization principles on polymer and polymeric delivery of nucleic acid vaccines in vivo.

### 4.1 Optimization of polymers

The implementation of the diversity and versatility of polymers using simple and efficient synthesis or modification methods is effective to improve the efficiency of nucleic acid vaccines. Currently, multiple strategies to improve the delivery efficiency of polymers have been developed, including (1) customizing polymer structures, molecular weights, and charge densities; (2) regulating the hydrophilic and hydrophobic properties of polymers; and (3) introducing appropriate adjuvant properties.

#### 4.1.1 Optimization of polymer structures

Polymeric delivery systems form complexes or physically encapsulate nucleic acids into NPs or microparticles to provide greater protection from nucleases, allowing for tunable degradation and controlled release, and facilitating modification to achieve cell-specific targeted delivery 137. The characteristics of polymers depend on the type, quantity, and connection form of monomers that constitute their structure. Polymers may be combined with other components or crosslinkers via physical (self-assembly) or chemical (covalent bonding) methods to produce customizable spatial features, including linear structures, dendritic macromolecular structures, constellations, stars, graft architectures, and networks. Wafa et al. synthesized three different polyanhydride copolymer compositions (50:50 CPTEG: CPH, 20:80 CPTEG: CPH, and 20:80 CPH: SA) via melt polycondensation. The hydrophobicity of polymers obtained by different condensation ratios is different, resulting in a decrease in the degradation rate, indicating that the polymeric structure affects the intensity of the CD8^+^ T cell response and increases the protective duration 138.

Linear polymers with amino groups that will be protonated at physiological pH were one of the first polymers used for nucleic acid delivery. Branched polymers and dendrimers with tunable molecular weights, formed by the diffusion of several branches along the backbone or core, are also widely studied as nucleic acid delivery vehicles. Their structures have many sites for structural modification, where different types of functional groups may be introduced at branch points, main chains, and end groups to achieve different delivery characteristics. However, simple linear and branched polymeric carriers have significant drawbacks related to molecular weight-dependent cytotoxicity 139. Graft copolymers including PEG-b-PEI, oligoamines, and oligopeptide combs, display low cytotoxicity and good transfection efficiency, are intensively investigated as promising carriers for nucleic acid vaccines. Biodegradable polycations have been newly developed, including poly (2-(acrylamido) glucopyranose), PBAE, poly (2-aminoethyl propylene phosphate), and degradable PEI, which show lower cytotoxicity and equivalent or higher transfection efficiency 140, 141.

APCs may be transfected directly by nucleic acid vaccines. Effective delivery of antigens to APCs through different structural polymers containing antigens to achieve target APCs and regulate antigen presentation is a logical choice to enhance the immunogenicity of nucleic acid vaccines 142. On one hand, this promotes cell uptake by targeting surface molecules of APCs, CD11c, DEC205, mannose receptor, DC-specific intercellular adhesion molecule-3-grabbing non-integrin, and TLRs, among others; on the other hand, this facilitates intracellular stimulation responses, including (1) an endogenous stimulation response involving pH, enzyme activity, and glutathione and (2) exogenous stimulation response, such as light irradiation, ultrasound, and magnetic fields 76.

The properties of different functional groups may be introduced to polymers through block synthesis and end-group modification. Introduction of hydrophilic or hydrophobic end groups, installation of responsive stimuli, and specific end group modification may increase transfection efficiency. However, vector toxicity needs to be taken into consideration during the polymer structure optimization.

#### 4.1.2 Optimization of the molecular weight of polymers

Blakney and co-workers used the optimized aza-Michael addition synthesis to obtain a bioreducible, linear, cationic polymer (5-167 kDa) for saRNA delivery. In vivo and in vitro experiments showed that increasing its molecular weight enhanced the delivery efficiency 66. Biomaterials used as a matrix for dissolving microneedles may affect the immunogenicity of incorporated vaccine antigens. Hyaluronic acid with a molecular weight of less than 150 kDa does not affect the antibody response, nor does it affect the CD4^+^ T cell response against model antigen ovalbumin 143. Mouse immune experiments carried out on hyaluronic acid with different molecular weights revealed that a 20-kDa hyaluronic acid microneedle was stable and dissolved rapidly in the skin without affecting immunogenicity 143. The molecular weight of polymers must be cross-checked to regulate the delivery efficiency and safety. A low molecular weight polymer has lower cytotoxicity and is beneficial to the unpacking and release of nucleic acids, but the transfection efficiency is not ideal. Increasing the molecular weight will lead to increased cytotoxicity. The range of molecular weight of different types of polymer vectors plays a key role in regulating gene transfection efficiency and vector cytotoxicity 144.

#### 4.1.3 Optimization of the charge density of polymers

Cationic amines containing polymers are used to form complexes with anionic plasmid macromolecules to facilitate their cellular uptake and inhibit plasmid degradation during extracellular and intracellular trafficking. Among them, the amino cations determine the charge density of the polymer matrices, and thus are the key to the carrier's delivery characteristics. The pKa of polymers is closely linked to the protonation of amino groups and may be used to improve the transfection efficiency. The toxicity and transfection efficiency of polymers with alkyl substituted amines are correlated with the type of amine (primary, secondary, and tertiary) 145. Therefore, the number of amino groups, type of substituents, and spatial arrangement of amino groups may have important effects on the value of pKa and delivery efficiency of polymer carriers. At present, the relationship between charge density and carrier presentation efficiency is not completely clear. The change in charge is mostly reflected in the change in the number of amino groups. Wei et al. synthesized poly(glycidyl methacrylate) (P(GMA)) homopolymers via reversible addition-fragmentation chain-transfer polymerization; and then modified them with different oligoamines to form tetraethylenepentamine, pentaethylenehexamine, and tris(2-aminoethyl)amine. The effects of the P(GMA) skeleton length and oligoamine structure on gene transfer efficiency were investigated and found that polymer P(GMA-TEPA)_50_ showed higher gene transfer efficiency 146.

#### 4.1.4 Optimization of the hydrophilicity and hydrophobicity of polymers

Regulating the hydrophilicity or hydrophobicity of polymers is an effective method to improve delivery efficiency and complex stability. The improvement in hydrophilicity is more reflected in improving the stability of carrier systems, avoiding self-aggregated particles and proteins adhesion in serum during circulation, and delaying the recognition and elimination by the immune systems. PEG, which forms a dense cloud of hydration on the surface of polyplexes and inhibits the adhesion and aggregation of proteins, is the most used polymer to adjust the hydrophilicity of a system by introducing hydrophilic components at the chain end or through grafting. Better protection of nucleic acids may be achieved by PEGylation, however, the performance of cell-internalized carrier systems may be reduced. Zwitterionic components, composed of equal amounts of positively and negatively charged ions, can form a dense cloud of hydration and are another widely studied hydrophilic material with good biocompatibility. Carbohydrates, such as glucose and trehalose, may also provide good hydrophilicity and stability for polymer delivery systems, enhancing the efficiency of nucleic acid delivery and significantly reducing cytotoxicity 147. The introduction of hydrophilic components may also help achieve an overall improvement in carrier systems in the circulation, while an improvement in hydrophobicity may focus on the fine-tuning of carrier systems.

Hydrophobicity is one of the major factors in modifying and optimizing nonviral gene delivery systems. Under the premise of sufficient water solubility and stability of the vector system, increasing the hydrophobicity of the polymer carrier may significantly improve the transfection efficiency of nucleic acids while reducing cell toxicity. Analyses concerning the interaction of cell membranes with amphiphilic materials have shown that the adsorption of hydrophobic moieties (i.e., more carbon atoms in the polymer backbone or side chains) may lead to disruption or destabilization of the cell membrane, thereby facilitating cellular uptake 148, 149. Therefore, cellular uptake is often promoted by increasing the hydrophobicity of polymers that interact between NPs and the cell membrane, leading to increased presentation efficiency of the carriers 150. In addition, the end groups may be fluorinated, alkylated, or acylated to optimize the hydrophobicity of a carrier system 151.

Delivery systems require an appropriate balance between hydrophilicity and hydrophobicity to achieve optimal delivery and transfection efficiency. Overall, the current methods of regulating the hydrophilicity and hydrophobicity of polymers are more inclined to the modification of the structure. The influence of the density and space structure of a graft on the carrier is worth exploring. The modification method of the structure needs to be simple, easy to operate, stable, and repeatable.

#### 4.1.5 Immunoadjuvant properties of polymers

Due to the limitations in the efficiency of nucleic acid vaccines, it is often difficult to induce strong and long-acting immune responses using translated antigens. Therefore, adjuvants have become one of the common methods to enhance immune responses and improve the efficacy of nucleic acid vaccines. Traditional vaccine adjuvants may be used to increase the immunogenicity of less immunogenic antigens; however, in general, the impact on DNA vaccine immunogenicity is at best modest, and attempts need be made to develop more effective adjuvant approaches 152. Alum, which has been widely used as a vaccine adjuvant since 1926, increases the antibody response of nucleic acid vaccines in a variety of animals and improves the protection rate of the *Toxoplasma gondii* vaccine 153. However, alum is mainly conducive to the Th2 immune response and may not be suitable for DNA vaccines that require CD8^+^ T cellular immune responses 154. As alternatives to alum, polysaccharide adjuvants based on β-inulin granules and Zymosan polysaccharides significantly increase humoral and cellular responses in mice when used in a DNA primary immunization protein-enhanced vaccine strategy 155. Another traditional adjuvant category includes oil emulsions, which improve the immunogenicity of the HIV-1 DNA vaccine moderately when mixed with plasmids 156.

Commonly used nano adjuvants include liposomes and polymeric NPs. Liposomes penetrate the lipid bilayer of the cell membrane, promote DNA entry into cells, and enhance cellular and humoral immunity. Like liposomes, polymeric NPs, such as chitosan, PEI, PAMAM, poly(D, L-lactide-co-glycolide) (PLG), and PLGA particles, protect plasmids from degradation and increase the uptake of cells 157. As previously mentioned, natural polymer chitosan has been successfully used for oral DNA vaccinations against *T. gondii*, *S. mansoni*, and Coxsackie B virus-induced myocarditis 25, 158. Hou et al. noticed that the co-inoculation of Cholera toxin B subunit (CTB) and HIV Env DNA vaccine in vivo enhanced the ENV-specific interferon (IFN)-γ HIV cellular immune response and promoted antibody maturation by activating the high-level expression of Th1 and Th2 cytokines and inflammatory cytokines and chemokines in mouse BMDCs through TLR signaling pathways, indicating that CTB may be used as an effective candidate adjuvant for DNA vaccines 159. The co-inoculation of low-viscosity carboxymethylcellulose sodium salt and (HIV-1) IIIB rev genes enhanced HIV-specific mucosal antibody (Ab) and systemic Ab and cell-mediated immune responses 160. Carroll et al. found that the cationic polysaccharide chitosan promoted DC activation by inducing type I IFNs and enhanced antigen-specific Th1 responses in a type I IFN receptor-dependent manner 161. In addition, the charge intensity and particle size are also key to the effect of immunization, and the appropriate charge intensity and particle size may improve the delivery efficiency of nucleic acids 162. Increasing the efficiency of immune activation and the possibility of quantitative production through vector integration with adjuvants are trends in vaccine development.

However, in general, the impact of traditional adjuvants and immune-enhancing polymer adjuvants on the immunogenicity of DNA vaccines is limited, which leads to attempts to develop more effective molecular adjuvant methods. Although these synthetic and natural polymer adjuvanted DNA vaccine delivery systems hold great promise, the instability of the polymeric system coupled with the variability in the physiological environment urges the development of better delivery strategies.

### 4.2 Optimization of immunization routes

**Figure 12:**
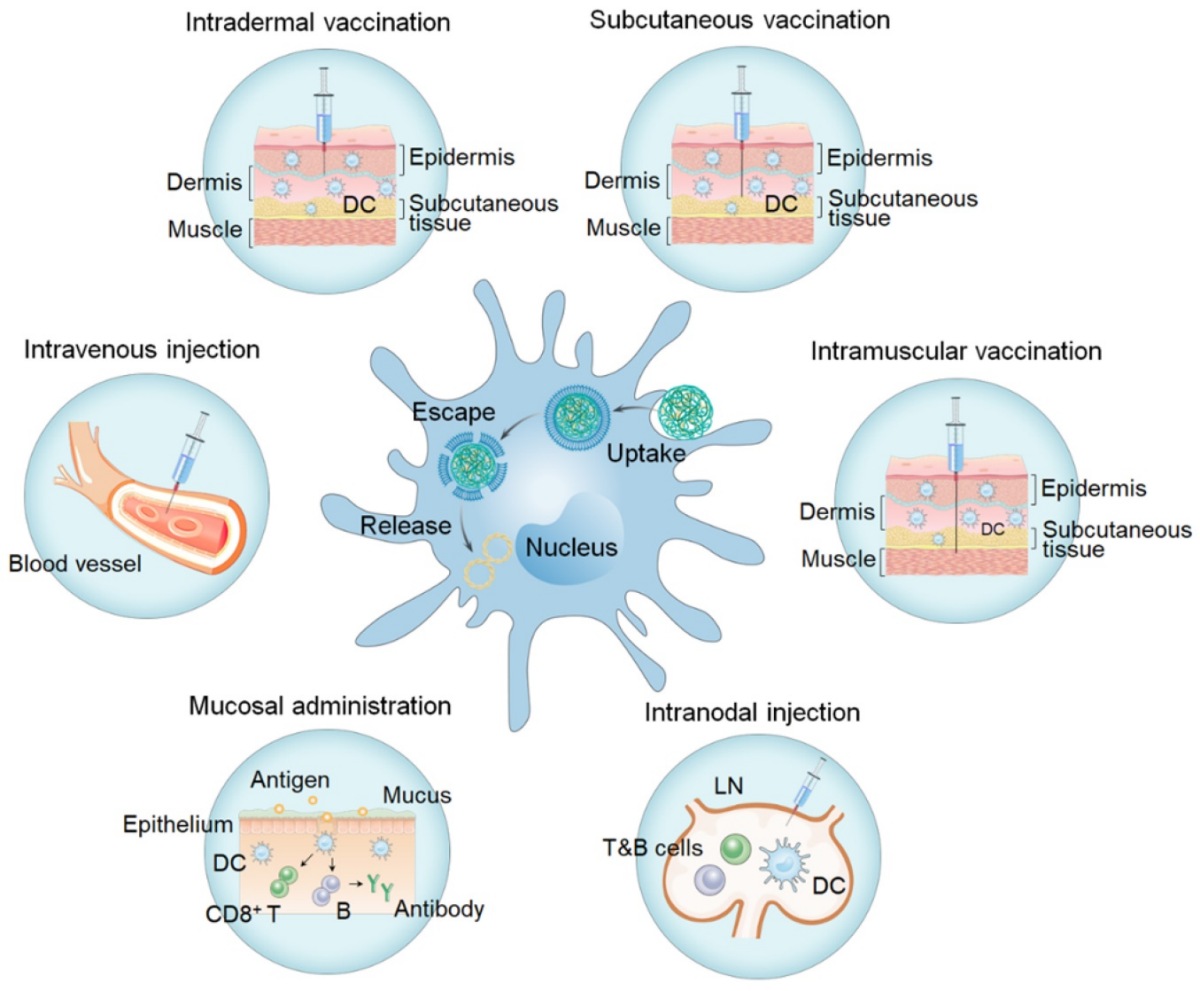
Different immunization routes (i.e. intradermal vaccination, subcutaneous vaccination, intramuscular vaccination, intravenous injection, mucosal administration, and intranodal injection) and APC (in the middle) uptake.

The main routes for vaccinations (Figure 12, Table 3) include transdermal administration (i.e., intradermal administration, subcutaneous administration), intramuscular vaccination, intravenous injection, mucosal administration, and LN injections. Activation of the immune response depends on vaccine uptake and antigen presentation by APCs, which are affected by the site of administration. Selection of the appropriate immunization route is crucial in determining the type and degree of the induced immune responses.

#### 4.2.1 Intradermal administration

The skin, one of the largest immune organs, is mainly composed of the epidermal layer and dermis and is rich in APCs such as DCs and macrophages for initiating adaptive immune responses. DCs are of great importance in skin immunity and serve as the key APCs to induce the activation of initial T cells. Langerhans cells are a specialized population of DCs, which extensively exist in the epidermis of the skin 175. Intradermal vaccination is a form of administration between the dermis and epidermis wherein nucleic acid uptake is accomplished mainly in three forms: dermal fibroblast uptake, Langerhans cell uptake, and drainage into LNs and uptake by resident DCs. After being taken up by DCs, and the antigens will be expressed in the cytoplasm. Specific epitopes are then presented on MHC-I and -II of DCs. DCs differentiate into mature DCs when stimulated by antigens or inflammatory mediators. Moreover, antigens are delivered to the initial CD4^+^ and CD8^+^ T cells in the local LN, and then T cells are effectively activated and differentiated with the assistance of costimulatory signals and cytokines to promote the occurrence of immune responses. Some polymers, such as PEI, chitosan, pABOL, poly(2-aminoethyl methacrylate), etc has been used for delivery of mRNA or DNA vaccines through intradermal injection 66, 87, 164, 176. In addition to the particles alone, polyplexes encapsulated in microneedles or injectable hydrogels have shown to be appropriate formulations for intradermal vaccination (Table 3) 52, 95, 96, 99, 107-109. Intradermal vaccinations overcome the barrier of the cuticle, greatly improving the nucleic acid uptake efficiency of APCs in the skin by controlling the exposure of nucleic acids and promoting the transport of antigens to draining LNs and inducing a stronger specific immune response with fewer side effects. Therefore, it is widely considered as an excellent route of vaccination.

#### 4.2.2 Subcutaneous administration

Subcutaneous tissue refers to loose connective tissue below the dermis, where some monocytes and DCs exist. Subcutaneous administration is another common route of vaccination. Nucleic acid is transported to the subcutaneous space and is taken up, processed, presented, and then migrated to local LNs through the lymphoid channel to activate T cells and B cells to trigger the immune response. Cui et al. delivered block/homo-mixed polyplex micelles carrying genes encoding tumor-associated antigen SART3, as well as CD40L and GM-CSF, into mice with peritoneally disseminated CT26 cancer via subcutaneous, intraperitoneal, intravenous, and electroporation methods 177. The results showed that compared with the three other methods, mice in the subcutaneous administration group had the highest survival rate with no abnormalities found at the local injection sites, in body weights, and in blood test results, demonstrating the possibility for subcutaneous immune pathways in tumor therapy. The hydrogel delivery system not only effectively enhances adhesion at the injection site but also does not cause inflammation. This in situ storage form is suitable for the delivery and transfection of nucleic acids. Le et al. formed a hydrogel reservoir via subcutaneous injection of PEG-modified poly(sulfamethazine ester urethane) sol on the dorsal side of Sprague Dawley rats and observed that the hydrogel reservoir could be effectively released in situ for more than 2 months 178. Skin, the largest immune organ of the human body, induces an immune response equivalent to that of other vaccination methods efficiently with a relatively low dose, making it an ideal vaccination site. Subcutaneous injections are associated with mild local stimulation and good tolerance but this also leads to low levels of pro-inflammatory cytokines and chemokines, which should be taken into consideration.

#### 4.2.3 Intramuscular injection

The advantages of rich blood flow, excellent capacity, good diffusion, and a lasting immune effect make intramuscular injections the main choice of vaccination route. A polymer carrier further enhances the duration of an immune response by intramuscular injections via protecting nucleic acids from degradation and enhancing the particle retention time. Reddy and co-workers immunized guinea pigs through intramuscular injections with hand-foot-and-mouth virus expression plasmids (pVAC-1D) wrapped in PLG polymer. Compared with the naked DNA, pVAC-1D-PLG not only significantly increased gene expression but also induced higher levels of humoral and cellular immune responses. Quantitative PCR results also showed that the plasmid persisted and expressed antigens in guinea pig tissue for more than 1 year 165. Urello et al. modified PEG-poly(_L_-lysine) (PEG-PLL) with morpholine and niacin (MN) to improve the buffering effect of the carriers, enhancing the particle's ability to resist polyanion exchange, and prolonging blood circulation. In addition, after intramuscular injection, PEG-PLL (MN) NPs were continuously transfected and expressed antigens in muscle tissue at a higher level than that of PEG-PLL for more than 45 days 179. During intramuscular injection, nucleic acids or their nanocomplexes are primarily transfected into muscle cells or DCs present in normal skeletal muscle. Lu and co-workers utilized *β*-cyclodextrin-PEI (CP) conjugates to load mRNA 176. The CP-mRNA polyplexes immunized mice through intradermal, muscular, and subcutaneous pathways. The CP-mRNA nanocomplex resulted in specific IgG production during intradermal and intramuscular injections. In addition, intradermal and intramuscular injections showed differences in antibody subtypes. Intramuscular injections induced IgG1 and IgG2a antibodies, while intradermal injections mainly induced IgG2a antibodies. These results indicate that both intramuscular and intradermal CP-mRNA polyplexes induce Th1 and Th2 immune responses but the former tends to Th2-type responses, while the latter shows Th1 orientation.

Abundant muscle cells express a variety of pro-inflammatory cytokines and chemokines under inflammatory stimuli, which helps maintain local inflammation and coordinate the recruitment of monocytes and DCs to promote nucleic acid uptake and antigen presentation. Myoblasts in muscle tissue also contribute to driving muscle-directed immune protection and may become facultative APCs in MHC-I- and MHC-II-dependent immune responses, forming functional immune synapses with T cells 180. TLRs are the pattern recognition receptors of pathogen-associated molecular patterns, which play an important role in inducing acquired and innate immunity in muscle vaccination. Skeletal muscle cells express multiple TLRs that promote the production of pro-inflammatory cytokines, enhance antigen presentation to naïve T cells, and activate antigen-specific immune responses.

#### 4.2.4 Intravenous injection

After intravenous injection, nucleic acids are rapidly transported in the blood circulation to the LNs and spleen, which are the center of immune activation and response to exogenous antigens. The spleen is an important lymphatic organ, which is rich in APCs, T cells, and natural killer cells, making it the main immune site for intravenous immunity. Fornaguera et al. developed a mRNA delivery system based on oligopeptide-modified PBAEs. The so-called KH oligopeptide enables a specific APC targeting in the spleen after intravenous injection and then achieve efficient transfection of APCs in vivo 169. The systemic and circulatory nature of intravenous vaccination tends to make it more suitable for the long-term treatment of tumors and invasive diseases. Yang et al. designed a PLGA-core/lipid-shell hybrid nanosystem for mRNA antigen and adjuvant (gardiquimod) co-delivery. Effective mRNA expression in the spleen and strong antigen-specific immune response in vivo were observed by intravenous injection. Finally, the administration of nano vaccines is found to be beneficial for tumor growth inhibition 170. It is remarkable that Yu et al. studied a high-throughput library of 1200 functional polyesters to identify superior polymers for systemic mRNA delivery. The structure-activity relationships between alkyl side chains and in vivo delivery were illustrated and a series of polyplexes stabilized with Pluronic for delivery of mRNA to lungs and spleens of mice were identified. This study should be helpful for the development of next-generation vaccines and therapeutics 171. In addition, intravenous injections also induced immune tolerance via liver delivery. Liu et al. developed a strategy that targeted natural tolerogenic liver sinusoidal endothelial cells using stabilizing receptor ligand-modified PLGA NPs as delivery vector 168. This strategy produced regulatory T cells that significantly inhibited antigen-specific immune responses. Due to several physiological barriers in circulation as discussed in 3.3.1, intravenous injection of vaccine has not been regarded as the preferred choice. The design of polymer carriers should be focused specifically on the DNA or RNA protection, long cycle properties, APC targeting, and stability of the vector to achieve a strong and lasting immune response.

#### 4.2.5 Mucosal administration

The mucosal surface is one of the main sites in which pathogens infect the body, and mucosal vaccination can neutralize pathogens before they cause infection and effectively induce mucosal and systematic antigen-specific immune responses. The mucosal immune system is mainly composed of mucosa-associated lymphoid tissues formed by inducers of the respiratory tract, gastrointestinal tract, urogenital system, etc. The core of mucosal immunity is the migration of APCs from the mucosal induction site and recruitment of immune cells to the mucosal effector site, thus effectively inducing the memory of persistent B and T lymphocytes 181. Oral and intranasal administrations are the most widely studied pathways for inducing mucosal immunity.

Oral vaccination is one of the most well-tolerated vaccination approaches. The sublingual region and buccal mucosa, consisting of the epithelium and mucosa, are attractive sites for vaccination. A variety of DCs, including Langerhans cells and CD11b^+^ and CD11c^+^ DCs, are widely present in mucosal and epithelial tissues near the mouth. Oral vaccines effectively induce immune responses in the mucous membrane, surrounding tissue, and even distal mucosa, and their good tissue permeability and easy adsorption are suitable for the efficient delivery of macromolecules 182. However, the dilution and excretion of a vaccine by saliva flow limit the efficiency of vaccination in the sublingual area and buccal mucosa. Hervouet et al. found that after sublingual immunization, sublingual DCs could enter the bloodstream toward distant lymphatic organ delivery, while uptake of antigens from sublingual mucosa by antigenic DCs was also observed in distant LNs and the spleen 183. Further data showed that the activation of CD8^+^ T cells was not induced by sublingual lymphatic or vascular-diffused adhesive-type antigens, but by antigen-carrying DCs in distant LNs and the spleen in a time and dose-dependent manner. Curtis II et al. used a variety of pathways to deliver simian immunodeficiency virus (SIV)-expressing DNA into young rhesus monkeys and then examining the strength of induced immunogenicity 184. The mucosal oral/tonsillar or oral/sublingual immune tissues in mice successfully induced the production of split vaccine-specific T-cells, but the SIV-specific antibodies were not detected in saliva, feces, or plasma. These low antibody levels may be due to inefficient oral uptake of DNA, however, strong T cell responses were also observed in distal mucosal LNs.

The small intestine is an ideal site for oral vaccines due to its superior ability to recognize and ingest large molecules. In one study, intestinal mucosal DCs were divided into two subgroups: one group expressed CD103^+^ and played an important role in the induction of mucosal immune tolerance and secretory IgA^+^ B cell differentiation; the other group expressed CD11b^+^, which upregulated costimulatory molecule expression and promoted the differentiation of primary T cells into effector T cells, inducing humoral immunity 185. Together, these DCs modulated the immune response in intestinal mucosa. Oral vaccines are widely used in fish and chicken farming. Valero et al. developed an oral DNA vaccine of nodavirus with chitosan as the delivery vector (CP-pNNV), which induced strong cellular immunity 173. The expression of cell-mediated cytotoxicity (TCRB and CD8a) and genes related to the interferon pathway (*IFN*, *MX*, and *IFNG*) were significantly upregulated. Meanwhile, the CP-pNNV vaccine showed good protection against a nodavirus attack. Leya et al. also induced a mucosal immune response in *Labeo rohita* by immersion 172. DNA was loaded into a PLGA-chitosan vector to form NPs, and *L. rohita* was immunized. The results showed that DNA vaccines played a protective role by inducing humoral immunity. The unsuccessful attempt of Valero et al. to induce humoral immunity after inoculation may be related to specific cellular antiviral mechanisms involved in fish conservation. In addition, other factors such as animal size, dose, frequency, and adjuvants also affect the quality of protective immunity.

The high abundance of DCs in the nasal cavity makes it a good site for inoculation. Nasal vaccinations induce immune responses of both the nasal lymph tissues and remote mucosal sites, such as the respiratory and urogenital tracts. Intranasal vaccination is preferred for respiratory diseases. Zhao et al. found that an immune response was induced by promoting CD103^+^ DC activation and Th17/T follicular helper cell (Tfh) differentiation in mice after intranasal immunization through the injection of chitosan-DNA complexes, further promoting IgA class switch recombination of gut B cells 186. Meanwhile, chitosan-DNA induces CD4^+^ Th17 and Tfh responses in vitro and in vivo, which contributed to the generation of IgA. In addition, chitosan-DNA enhanced the migration of intestinal DCs to LNs and significantly promoted the differentiation of antigen-specific IgA^+^ plasma blasts and plasma cells, suggesting that intranasal immunization in mice may induce a strong immune response. Intranasal immunity has also been shown to work well in large animals. Souci et al. used PLGA-PEI as DNA encoding an immunogenic glycoprotein B (PrV-gB-based DNA) vector for intranasal immunization in pigs against pseudorabies virus (PrV) 187. After intranasal inoculation, induction of PrV in pig serum-specific IgG and IgA antibodies, and muscle injections of mucosal salivary IgA antibodies against PrV. PLGA-PEI particles were found to significantly prolong the duration of IgA production compared with naked DNA. This suggests that the intranasal form of vaccination is effective in preventing respiratory disease in pigs.

The mucous layer is a dynamic barrier secreted by epithelial cells, covering the surface of the epithelium 188. It protects the mucosa from pathogen invasion but also seriously hinders the mucosal route of vaccine delivery. After entering the mucus, some vaccine particles interact with proteins in the mucus and are captured by mucus fibers before being whisked out of the body in a rapid turnover of mucus. The abundance of carboxyl and sulfate groups and sialic acid in the mucus confer a negative charge, affecting the diffusion of vaccine particles 181. Vaccine particles must cross the barrier of the mucus layer and epithelial cells and then be taken up by APCs, which significantly reduces the immune effect. Prolonging the residence time of particles at the mucosal site substantially improves the uptake of nucleic acids and antigen presentation efficiency. Therefore, it is of great significance for mucosal immunity to design intelligent polymer delivery carriers according to the thickness, pH, protein concentration, and related rheological properties of mucous layers at different sites.

#### 4.2.6 Intranodal injection

Intra-LN injection directly target APC-rich environments by enriching vaccine components in local LNs, simplifying the migration process of DCs, improving the nucleic acid uptake efficiency of LN-resident DCs in a short period, and inducing a rapid and powerful immune response 189. Intranodal injections not only maximize the dose response but also improve the quality of the immune response, selectively promoting Th1-biased cytokines and a humoral response 190. Regulation of the residence of nucleic acids in the LN region by polymer carriers can significantly increase the immune response. Jewell et al. injected fluorescently labeled PLGA microparticles into the inguinal LNs of mice and observed strong and local fluorescence signals in the inguinal LN region 174. Histological analysis also showed that microparticles were mainly located in the LNs. Intranodal and intramuscular injections were then administered with microparticle-loaded TLR-3 ligand poly (inosinic: cytidylic acid) (Poly(I:C)). Twenty-four hours after intranodal injection, histological analysis revealed that the particles had localized in the LNs, but this was not observed in mice that received intramuscular injections. Intranodal injections increased particle uptake by DCs, macrophages, and B cells by 8-, 10-, and 13-fold, respectively, which contributed to Poly(I:C) accumulation and sustained DC activation in LN-resident APCs. Intranodal injections represent the most direct manner to deliver vaccines to LNs. However, they usually require surgery or complex operations, which are typically associated with some risks 78. Furthermore, relatively small LNs may restrict the injection volume, limiting the administration of the optimal dose. Therefore, intranodal injections may not be a suitable immunization route for widespread application.

### 4.3 Co-delivery and combination therapy

To address the main issue of low genetic vaccine immunogenicity, the co-delivery of antigen-encoding nucleic acids and drugs, for example, antigen-encoding pDNA and immune-modulatory molecules, is a critical research area toward gene-based immunotherapy and vaccines. A novel star-shaped polymeric nanocarrier for the co-delivery of pDNA and imiquimod, a poorly soluble small molecule adjuvant, to DCs was developed by Lin et al. The co-delivery showed higher transfection efficiency on in vitro transfection assays, with potential use in genetic vaccine approaches 191. Self-assembled intertwining DNA-RNA nano capsules achieved the effective delivery of synergistic DNA CpG and short-hairpin RNA adjuvants, as well as tumor-specific peptide neoantigens, and triggered 8-fold more neoantigen-specific peripheral CD8^+^ T cells than CPG 192. Nucleic acid vaccines and Evans blue were combined as albumin-binding vaccines (AlbiVax) to form albumin/AlbiVax nanocomplexes. After the self-assembly of nanocomposites with endogenous albumin in vivo, a composite delivery system was formed. Compared with the benchmark incomplete Freund's adjuvant, the composite NPs show a 100-fold higher delivery efficiency (tending to LNs), trigger a 10-fold greater peripheral antigen-specific CD8^+^ immune memory, demonstrate effective vaccine delivery and effective cancer immunotherapy, and were regarded as a powerful platform for cancer immunotherapy 193.

TLRs recognize structurally conserved molecules derived from microorganisms and play a key role in the activation of the innate immune system. Among them, the ligands of TLR3 and TLR9 may be used as molecular adjuvants because they recognize double-stranded RNA and single-stranded DNA, respectively. The ligand Poly(I:C) of TLR3 and ligand CpG of TLR9 have been successfully used to enhance the immune response of DNA vaccines against pathogens such as tumors and human papillomavirus-16 162. Similarly, RIG-I and MDA5, as receptors for viral RNA, replication intermediates or transcripts, are also potential molecular adjuvants. RIG-I agonist eRNA41H may be used to enhance humoral immunity against influenza induced by DNA vaccines 194. Cytokines are naturally secreted small proteins that mediate immune signals. Cytokines that may be used as molecular regulatory adjuvants of nucleic acid vaccines include IL-2, IL-12, IL-15, and GM-CSF, which activate innate immunity in response to type I IFN produced by TLR signals, enhancing adaptive immune responses and improving the immunogenicity of DNA vaccines 195. Cytokines share expression plasmids with antigens, which has the advantages of a simple design, low cost, and easy control. Chemokines bind to G protein-coupled surface receptors to regulate leukocyte transport. The transfection of chemokine expression vectors with DNA vaccines helps enhance T cell activation and augment the CTL response of nucleic acid vaccines. Adjuvant signaling molecules, including IFN regulatory factor (IRF), programmed death-1, macrophage inflammatory protein (MIP)-1α, MIP-3α, MIP-3β, RANTES, IFN-γ-inducible protein-10, and CCR7, have become possible strategies to improve nucleic acid vaccines 162. Compared to cytokines, chemokines are more stable and have less potential for inflammatory toxicity and may therefore serve as better nucleic acid vaccine adjuvant candidates 196. In addition, costimulatory molecules such as CD28 and CD40 have also been used as molecular adjuvants for nucleic acid vaccines, which play a key role in the interaction between innate and adaptive immune cells and represent promising nucleic acid vaccine adjuvants 197. Immune signaling molecules IRF3, IRF7, high mobility group box-1, and heat-shock protein 70 induce innate immune responses, followed by signal transduction through TIR-domain-containing adapter-inducing IFN-β- or MyD88-dependent pathways, resulting in the activation of key transcription factors that activate the immune response of nucleic acid vaccines. This approach has been applied in HIV-1 Gag, Env, and influenza nucleic acid vaccines 198. Although the research data related to the use of vaccine molecular adjuvants are limited, it seems to be a promising direction to fine-tune the immune response to nucleic acid vaccines.

Overall, because co-delivery and combination therapy play a key role in the interactions between innate and adaptive immune routes, they represent highly promising DNA vaccine enhancement strategies. The clinical application of co-delivery systems also needs to consider the balance between manufacturing cost and practicability in individualized treatment. Compared with single vaccines, the storage stability and safety of co-delivery systems have not yet been fully studied.

## 5. Conclusion

Polymers for nucleic acid delivery have been extensively explored in various biomedical applications. We have observed great progress in the development of polymers that exhibit great potentials toward the delivery of nucleic acid vaccines for the prevention and treatment of various diseases, such as infections, cancer, and autoimmunity. In the last several years, we have seen the rapid development of artificial intelligence and highly computerized technology, improving our analysis of genetic variation of pathogens and tumors toward novel and potent DNA or RNA vaccines. Innovative methods have been developed to synthesize and modify various functional polymers. Integration of adjuvant activity into the polymer carriers may be an effective approach for amplifying the immune responses during vaccine delivery. Novel systems, such as NPs or macroscale formulations, have been designed for nucleic acid vaccine delivery. The ultimate goal of vaccination is building effective immune memory rather than the temporary immune response, therefore, rational design of nucleic acid vaccine and their polymeric carrier should be worth heeding, and thorough assessment in vitro and in vivo is needed. The physiological principles and properties of polymers deserve to be further investigated. Despite polymeric delivery of nucleic acid vaccines has progressed significantly in preclinical studies, unlike lipid-based carriers, only PEI and PLGA reached clinical trials. The success of lipid carriers provides the principles for the development of polymeric vaccine delivery. Polymers are known to offer advantages over lipids when considering versatility, tunability, and scalability. However, their clinical translations as vaccine candidates and ultimately to the market require comprehensive safety evaluation of the polymers and their degradation products and satisfactory quality controls in compliance with the complexity of the polymeric structures. We hope that this review serves as a practical guide in the application of polymeric materials for nucleic acid vaccine delivery.

## Figures and Tables

**Table 1:** General effects of size, surface charge, and rigidity on LN distribution, LN retention, DC uptake, and DC maturation of particulate vaccines. “-” means no related reports.

	LN distribution	LN retention	DC uptake	DC maturation	Intracellular localization
**Size**	•10~200 nm particles can traffic into the lymphatic capillary and then reach LN. •Particles larger than 200 nm can be phagocytosed by peripheral DCs and be transported to LN.	Large particles show good retention in LN.	Large particles taken up by DC are higher than small ones.	DC activation is negatively associated with the size of particles.	Small particles are more efficient in endolysosomal escape and cytoplasm localization than large ones.
**Surface charge**	Positively charged particles may be trapped by glycosaminoglycans and proteins in tissue interstitium and fluid, resulting in failing to drain into LN.	-	Positive charges are beneficial to DC uptake than negative or neutral charges.	Positive charges facilitate DC maturation.	Positive charges promote endlysosome escape and cytoplasm distribution.
**Rigidity**	It is dependent on administration routes.	-	Rigid particles are more likely to be taken up by DCs.	Rigid particles facilitate DC maturation.	-

**Table 2:** The physiological barriers during nucleic acid vaccine delivery in vivo

	Delivery targets	Delivery barriers	Resulting impacts	Ref.
The delivery barriers at the organism level	LN and spleen	Protein adsorption, salt enviroments, enzymes, reticuloendothelial system, off-target effects	Particle size increase, formation of “protein corona”, aggregation, nucleic acid degradation, rapid elimination, side effects	121, 122
The delivery barriers at the organ/tissue level	LN	Uptake by local APCs, poor LN-targeted capacity, the trap of the extracellular matrix	Generation of immune tolerance against encoded protein, poor LN delivery efficiency	76, 132
	Spleen	Interaction with proteins in circulation, interaction with erythrocytes, the uptake by innate immune cells	Dissociation or aggregation of the delivery system, the vaccine delivery to non-target organs, rapid elimination of nucleic acid vaccines, undesired activation of innate immunity, formation of inflammation	7, 125, 133
	Nasal-associated lymphoid tissue	Mucus layer, mucosal epithelia	Rapid elimination of nucleic acid vaccines	7, 134
	Gut-associated lymphoid tissue	Extremely acid environment in the stomach, the intestinal microbes and nuclease	Degradation of nucleic acid vaccines	7
The delivery barriers at the cellular level	Cell membrane	The size restrictions of transmembrane pores and channels, low partition coefficients of cell membrane	Poor cellular entry efficiency	128, 129, 135
	Cytoplasm region	The acidic environment and nuclease in endolysosome, strong ionic interaction between cationic polymers and payload nucleic acid vaccines	Degradation of nucleic acid vaccines, poor transfection efficiency	119, 130, 136
	Nucleus	Nuclear membranes, NPC	Limited nuclear transport	7, 131, 135

**Table 3:** Summary of advantages and disadvantages of different immunization routes

Immunization routes	Advantages	Disadvantages	Ref.
Transdermal administration	Large quantities of APCs, avoiding the first pass effect	Higher incidence of local reactogenicity including primarily mild pain, swelling, and redness	52, 66, 96, 99, 106-109, 163, 164
Intramuscular vaccination	Large capacity, good diffusion, safety, convenience, long-lasting immunity	Lack of sufficient APCs	66, 93, 165-167
Intravenous injection	Rapid distribution to the immune organs along with the blood circulation	Interference of particles stability in circulation, rapid clearance by the mononuclear phagocytic system, leading to acute inflammation and severe acute renal and hepatotoxicity, need of high dose due to the off-target effects	168-171
Mucosal administration	High degree of mucosal immunity, needle-free delivery route, high patient compliance, avoiding the first pass effect	Obstruction and clearance by mucus layers, obstruction by mucosal epithelia	172, 173
Intranodal injection	Large quantities of APCs, T cells, and B cells, improving nucleic acid uptake efficiency	Complex and difficult operation, limited injection volume, small particles facing poor LN retention	174
